# Polymer Informatics Method for Fast and Accurate Prediction of the Glass Transition Temperature from Chemical Structure

**DOI:** 10.1021/acs.macromol.5c00178

**Published:** 2025-06-16

**Authors:** Sebastian Brierley-Croft, Peter D. Olmsted, Peter J. Hine, Richard J. Mandle, Adam Chaplin, John Grasmeder, Johan Mattsson

**Affiliations:** † School of Physics and Astronomy, 4468 University of Leeds, Leeds LS2 9JT, U.K.; ‡ School of Chemistry, 8368 University of Leeds, Leeds LS2 9JT, U.K.; § Department of Physics and Institute for Soft Matter Synthesis and Metrology, Georgetown University, Washington, D.C. 20057, United States; ∥ 120986 Victrex PLC, Hillhouse International, Thornton Cleveleys, Lancashire FY5 4 QD, U.K.

## Abstract

We present a new polymer informatics framework that successfully predicts the glass transition temperature *T*_g_ of polymers based on their chemical structure. The framework combines ideas from group additive properties (GAP) and quantitative structure–property relationship (QSPR) methods, where GAP (or group contributions) assumes that submonomer motifs contribute additively to *T*_g_, and QSPR links *T*_g_ to the physicochemical properties of the structure through a set of molecular descriptors. By integrating these methodologies, our combined QSPR–GAP framework overcomes limitations inherent in using either method independently. We demonstrate its application on a data set of 146 linear homo- and copolymers of the poly­(aryl ether ketone) (PAEK) family, achieving a median root mean square error of 8 K for *T*_g_, representing a significant improvement over standalone QSPR or GAP models. Moreover, using a genetic algorithm, we identify two molecular descriptors that predominantly drive *T*_g_ predictions. The QSPR–GAP framework can be readily adapted to forecast other physical properties and activity (QSAR) or transferred to other polymer families, including conjugated and biopolymers.

## Graphical abstract

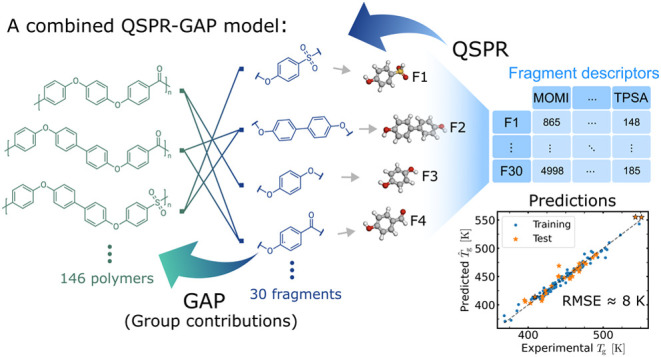

## Introduction

Polymers are remarkably versatile materials, and the combined control of monomer chemistry and chain length allows for superior tunability of physical properties. As a polymer melt is cooled, the timescale τ_α_ characterizing its structural (α) relaxation increases dramatically, and in the absence of crystallization, the structure freezes into an amorphous solid, a glass, at the glass transition temperature *T*_g_.^1^ Since molecular motions are controlled by *T*_g_, this is a key parameter for understanding and predicting material behavior, and it is thus essential to develop methods for accurately predicting *T*_g_ directly from the chemical structure.

For long-chain polymers, *T*_g_ is molecular weight (*M*)-independent^2–5^ but strongly affected both by intramolecular dihedral barriers^6,7^ (chain flexibility) and intermolecular packing effects, both of which are chemistry-specific.^5^ Importantly, it has been shown that the α relaxation, which defines *T*_g_, is linked to relaxations on a relatively ‘local’ submonomer length scale,^8–14^ which in turn suggests that models that predict *T*_g_ from monomer structure should be achievable. In this paper, we present such a model and apply it to the poly­(aryl ether ketone) (PAEK) family of polymers.

Predictive models that relate structure-based properties and *T*_g_ and are suitable for small data sets with low chemical variability have been proposed for polymers.^5,15–21^ For instance, an approximate correlation has been found between *T*_g_ and monomer-scale properties such as the molecular weight per conformational (or flexible) degree of freedom of the chain (*M*_ϕ_),^5,15–18^ where *M*_ϕ_ captures both chain flexibility and chain bulkiness (reflecting molecular packing). As one example, Schut et al.^18^ correlated *T*_g_ with the mass per flexible bond for a data set divided into three polymer classes by introducing flexible groups into both the main chain and the side chains; an out-of-sample mean absolute error (MAE) for the *T*_g_ of ≲ 6 K (per polymer class) was obtained. In another example, Xie et al.^19^ assigned an ad-hoc mobility factor to each atom based on the chemical group it belongs to (e.g., alkyl, phenyl, or thiophene). The monomer’s mobility was then averaged over the atomic contributions, followed by a regression of *T*_g_ on the monomer mobility. For a family of 32 conjugated polymers, an RMSE ≃ 13 K was attained for in-sample *T*_g_ predictions. These methods are easily applicable and intuitive, e.g., by linking a relevant physical property, such as molecular weight or volume, to each ‘flexible bond’, where ad-hoc rules are often introduced to quantify the influence of different bonds. However, the approaches are typically tailored to specific data sets and are not generalizable to a wider set of polymer structures.^20^

Conversely, a more generalizable approach is the so-called group contribution or the group additive properties (GAP) method.^22–24^ It assumes that a polymer property can be expressed by a composition-weighted average over contributions from submonomer motifs (fragments). The fragment contributions can be determined directly from the data by a linear regression. van Krevelen^24^ applied GAP to predict various polymer properties, such as transition temperatures; solubility; and mechanical, optical, and electrical properties, while Weyland et al.^23^ quoted in-sample MAE ≃ 10 K for predictions of *T*_g_. Despite their broad applicability, a fundamental flaw of GAP models is that they cannot be used to make predictions for polymers containing fragments outside of the data sample.^21,25,26^

A method that addresses some shortcomings of GAP models is the so-called quantitative structure–property relationship (QSPR) approach. QSPR-based methods use molecular descriptors,^27,28^ which quantify electronic, topological, or geometric properties that are calculated from atomistic representations of molecules. For polymers, QSPR methods are normally applied either to the monomer^21,25,29,30^ or to oligomers consisting of a few monomers,^31–33^ and statistical or machine learning (ML) techniques are used to determine the relationship between the descriptors and the investigated property (such as *T*_g_).^30,34,35^ For QSPR methods applied to *T*_g_ predictions, RMSEs typically vary from ≃4 to 35 K,^21,25,36–38^ depending on the chemical variation within the data set. Models on larger data sets,^39,40^ with higher chemical variation, typically yield prediction errors exceeding 25 K.^36,41^ A significant drawback of QSPR models is that accurate descriptor calculations can be computationally costly, especially for large monomers or oligomers.

GAP and QSPR methods have usually been applied separately.^19–21,29^ However, Hopfinger et al.^26^ proposed a linear regression-based model for predicting *T*_g_ based on a GAP-like averaging scheme, combined with associating physical properties (conformational entropy and mass) with individual bonds. Inspired by this approach, we suggest extending QSPR methods to a smaller structural scale than the monomer unit, assuming interactions between these submonomer motifs negligibly contribute to the property of interest.

Here, we resolve the shortcomings of both the GAP and standard QSPR models by developing a hybrid QSPR–GAP method: a molecule is divided into submonomer fragments for which molecular descriptors are calculated, and various linear regression methods are used to link *T*_g_ to the fragment structure. The QSPR–GAP method provides more accurate predictions than either of the standalone methods, significantly faster descriptor calculations compared with QSPR, and accurate predictions of polymers containing fragments *outside* of the training set (where GAP fails).

We apply our new QSPR–GAP method to a data set of 146 linear homo- and copolymers of poly­(aryl ether ketone) (PAEK)—an important class of linear polymers characterized by alternating stiff (aryls such as phenyls or biphenyls) and flexible linker (such as ethers or ketones) moieties, as shown in Figure 1A. The properties of PAEK polymers are highly tunable by varying these moieties, making them suitable for a wide range of applications including smartphone speakers, electrical insulation, automotive gears, medical implants, and aircraft components.^42^ To design PAEK polymers with optimized properties for specific applications, reliable structure–property relationships are essential. Recent work^43^ investigated a similar class of polymers (poly­(aryl ethers)), predicting *T*_g_ using a purely QSPR-based approach where descriptors are calculated on the monomer units (referred to as “repeat units” by the authors), achieving an RMSE of ≃17–19 K.

**Figure 1:**
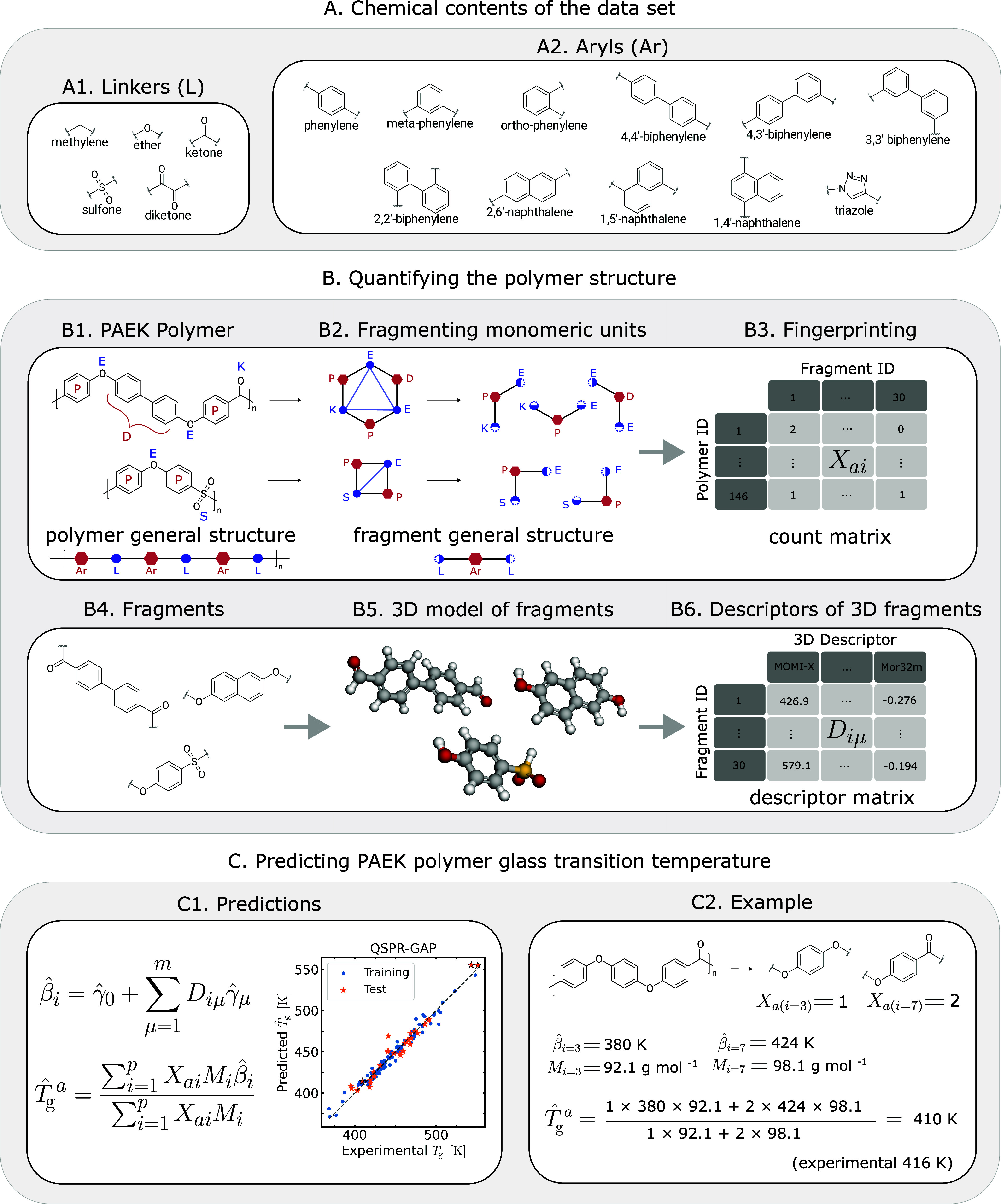
QSPR–GAP analysis: predicting *T*_g_ from the polymer structure. (A) Chemical building blocks (flexible linker and aryl moieties) of the PAEK polymer data set. (B) Quantifying the monomeric structure: a step-by-step illustration: Fragments are extracted from the repeating units while recording their occurrences in the monomer using a count-based fingerprinting scheme. 3D molecular models are generated from the fragments, and descriptors are computed from the 3D fragments. (C) Calculations required to obtain a predicted *T*_g_ from the descriptor and count matrix, where an example illustrates the latter calculation on poly­(ether ether ketone) (PEEK). Values of β̂_*i*_ and *M*_*i*_ are listed in Table S5, SI.

Our alternative QSPR–GAP method predicts *T*_g_ from the chemical structure with an RMSE of ≃5–12 K (out-of-sample). Moreover, by identifying the molecular descriptors most important for predicting *T*_g_, we reach new insights into how the local molecular structure relates to the glass transition temperature in polymers. Our findings offer a pathway to predict the properties of highly complex polymer structures using small data sets, thus circumventing the need for more elaborate ML methods that typically require larger data sets. Our method is readily generalizable to both a wider range of polymer properties (such as mechanical, optical, or electrical properties) and different classes of polymers.

## Results and Discussion

### Characterization of PAEK Polymers

Our QSPR–GAP model is applied to a data set of 77 PAEK homopolymers and 69 copolymers, sourced from both the literature and experimental measurements conducted by Victrex R&D. We ignore any minor effects of chain length on *T*_g_ ^5^ and assume that all measured *T*_g_ ≡ *T*_g_^∞^ (the long-chain limit); this limit is reached for PAEKs with *M*_*w*_ ≳ 25 kg/mol.^44^ We note that many of the PAEKs investigated are commercial-grade, and although we do not have supporting molecular weight data, the manufacturing process generally does not allow access to molecular weights lower than this limit.

The monomer of a PAEK polymer (see examples in Figure 1,B1) is a sequence of alternating rigid aryl *Ar* (Figure 1,A2) and flexible linker *L* (Figure 1,A1) moieties, where the alternating arrangement

1 $$...L_{1}-Ar_{1}-L_{2}-Ar_{2}...L_{N}-Ar_{N}...$$

is simple, yet different choices of *Ar* and *L* moieties lead to diverse material behavior, as illustrated by the *T*_g_ range of 375–550 K for the present polymer data set (Figure S1, SI).

We divide the monomer structure into unique submonomer “fragments” that constitute all PAEK monomers in the data set. Many fragment choices are possible, including *L–Ar*, *L–Ar–L*, *Ar–L–Ar*, or even longer sections. However, we mainly focus on *L–Ar–L* since the calculation of descriptors (see details below) requires the addition of hydrogens to the two ends of the fragments, and *L–Ar–L* is the only candidate that retains the uniqueness of the fragments once end-capped with hydrogens (Figure S20, SI). The data set of 146 polymers comprises 30 unique *L–Ar–L* fragments (Figure S3, SI), and as two examples, Figure 1,B1 illustrates how the monomers of poly­(ether sulfone) (PES) and poly­(ether biphenyl ether ketone) (PEDEK) are divided into *L–Ar–L* fragments.

We also benchmark QSPR–GAP against both the pure GAP and pure QSPR frameworks. For the GAP and QSPR–GAP methods, each homopolymer is parametrized by its *count matrix* **X**, where *X*_*ai*_ is the (integer) number of occurrences of fragment *i* in homopolymer *a*’s monomer (see the illustration in Figure 1,B3). Correspondingly, for copolymer *a*, we define *X*_*ai*_ = ∑_ξ = 1_^*l*^ *w*_ξ_ *X*_*ξai*_ , where *w*_ξ_ is the molecular weight fraction of comonomer ξ and *X*_*ξai*_ is the count of fragment *i* in copolymer *a*’s comonomer ξ.

For the QSPR–GAP and QSPR models, the molecular descriptors are calculated on the submonomer fragments and the monomer repeating units, respectively. Apart from the starting motifs (*i.e.*, the fragments vs the monomer repeat units), the procedure for calculating the descriptors is identical in both QSPR–GAP and QSPR frameworks: (i) add hydrogen atoms to the ends of each motif, (ii) generate an energy-minimized 3D representation of each motif (Figure 1,B5) using the Merck Molecular Force Field (MMFF)^45^ via RDKit,^46^ and (iii) calculate the values of molecular descriptors using Mordred.^47^ Using this procedure, we calculate *m* = 213 descriptors for every unique motif.

The descriptors consist of six types: 41 charged partial surface area (CPSA) descriptors, 4 geometrical indices, 4 gravitational indices,^27,28^ 160 3D-MoRSE descriptors,^48–50^ 3 moment of inertia descriptors, and 1 plane of best fit (PBF) descriptor.^51^ For the QSPR–GAP method, these μ = 1, 2, ..., *m* descriptors encode the *i* = 1, ..., *p* fragments constituting the *descriptor matrix* **D**, where *D*_*iμ*_ provides the value of descriptor *μ* for fragment *i* (see Figure 1,B6). The same *μ* = 1, 2, ..., *m* descriptors apply to the pure QSPR method, but now they encode the *a* = 1, ..., *A* polymers, thus constituting a polymer-based descriptor matrix, where *D*_*aμ*_ gives the value of descriptor *μ* for polymer *a*. In this case, we note that copolymer descriptors were obtained by *D*_*aμ*_ = ∑_ξ = 1_^*l*^ *w*_ξ_ *D*_*ξaμ*_ , where *D*_*ξaμ*_ is the descriptor value *μ* in copolymer *a*’s comonomer ξ. Finally, to maintain independence with the size of the repeating unit, all descriptors were normalized by the mass of the repeating unit (averaged for copolymers), resulting in the full set of inputs for the QSPR regression models (noting that this step only applies to the pure QSPR method).

The generation of 3D molecules and subsequent descriptor calculations are significantly faster for the QSPR–GAP method compared to the pure QSPR approach, with CPU times of a few seconds compared to 140 min. The speedup is primarily driven by two factors. First, the MMFF energy optimization requires fewer conformational degrees of freedom for the smaller fragment molecules compared to the entire (flexible) repeating monomer unit. Second, the QSPR–GAP method requires the energy optimization of 30 fragment molecules, whereas the QSPR method requires the energy optimization of 83 unique monomeric repeating units. For the full details on the 3D molecular optimization process; see SI Sec. S-IB.

### GAP and QSPR–GAP Approaches

The predicted glass transition temperature for the *a*th polymer *T̂*_g_^*a*^ is represented as a molar mass-weighted average of the estimated *T*_g_-contribution β̂_*i*_ from each *i*th fragment

2 $${\mathrm{T̂}}_{g}^{a}=\frac{\sum_{i=1}^{p}X_{\mathrm{ai}}M_{i}{\mathrm{\beta ̂}}_{i}}{\sum_{i=1}^{p}X_{\mathrm{ai}}M_{i}}\equiv \sum_{i=1}^{p}{\mathrm{X̅}}_{\mathrm{ai}}{\mathrm{\beta ̂}}_{i}$$

where *i* indexes the fragments (as labeled in Figure S3, SI), *M*_*i*_ is the molar mass of the *i*th fragment, and thus *X̅*_*ai*_ is the mass-weighted composition of fragment *i* in polymer *a*. The polymer *T*_g_ is thus modeled by its composition-weighted constituent fragment contributions, β_*i*_ , where β_*i*_ corresponds to *T*_g_ of a long-chain homopolymer composed entirely of the *i*th fragment. Since β_*i*_ is unknown, it is estimated; note that we denote an estimated (or predicted) value by a hat ^.

We estimate β_*i*_ in two different ways: (i) as a benchmark, we use a GAP approach based simply on the identity of the fragment or (ii) a novel combined QSPR–GAP approach based on the molecular features of each fragment encoded in the descriptors. In the GAP approach, the count matrix **X** is molar mass-normalized (eq 9), giving the composition matrix **X̅** with elements *X̅*_*ai*_, and β_*i*_ is estimated from the experimentally available *T*_g_ values by ordinary least-squares (OLS) regression against X̅ (eq 11).

In the QSPR–GAP approach, the key distinction from the GAP method lies in the parametrization of β_*i*_ (and consequently, *T*_g_) by a set of molecular descriptors that encode the structure of each fragment (see the Methods section for a detailed description of how β_*i*_ is estimated). The *T*_g_ contribution of fragment *i* is expressed in terms of the values of the molecular descriptors *D*_*iμ*_ , according to

3 $$\beta_{i}=\gamma_{0}+\sum_{\mu =1}^{m}D_{i\mu }\gamma_{\mu }$$

Here, the regression coefficient γ_μ_ parametrizes the influence of molecular descriptor μ on *T*_g_, and γ_0_ is a constant, both of which are estimated by the regression methods explained below. Since the inputs of eq 3 are physical molecular descriptors rather than occurrences of a given fragment, the QSPR–GAP model can also be used to predict the *T*_g_ value of polymers that contain a *j*th fragment that does not exist within the data sample.

### Regression Methods

Our data set of *n* = 146 polymers with corresponding *T*_g_ values was divided into *p* = 30 unique *L–Ar–L* fragments. The benchmark GAP analysis was performed using OLS to estimate the *T*_g_ contributions, β_*i*_, of fragments *i* = 1, ..., *p*. For the QSPR–GAP analysis, in turn, the information about the *p* = 30 fragments was encoded into *m* = 213 molecular descriptors, and four linear regression methods were used to determine γ̂_0_ and γ̂_μ_ (for each descriptor, μ = 1, ..., *m*): principal component regression (PCR), ridge regression, lasso regression,^52^ and partial least-squares (PLS) regression^53^ (see SI Sec. S–II for a brief discussion of each). The benchmark QSPR models were based on descriptors derived directly from the *n* = 146 polymers instead of from the *p* = 30 fragments. For this series of models, we applied the linear regression methods already mentioned and an additional nonlinear model: kernel ridge regression (KRR) with a radial basis function (RBF) kernel (SI Sec. S–II).

These regression methods were chosen due to their robustness against overfitting, which would otherwise occur since the number of fit parameters in eq 3 (*m* + 1) exceeds the number of observed data points (i.e., the *n* polymers). The regression methods also account for the multicolinearity among the molecular descriptors (see Figures S5 and S6, SI) by penalizing the size of the estimated coefficients γ̂_μ_, resulting in many fewer ‘effective’ regression coefficients.

As an alternative implementation of QSPR–GAP, a genetic algorithm (GA) was applied to select the subset of *m*_GA_ descriptors (out of all *m* = 213 descriptors) that best predict *T*_g_ by linear regression (see SI Sec. S–II for more details). Ten GA models were investigated, here termed “QSPR–GAP GA*m*_GA_” (*m*_GA_ = 1, ..., 10), each resulting in different estimates for coefficients γ_0_ and γ_μ_ = 1, ..., *m*_GA_, for the *m*_GA_ descriptors chosen.

### Performance of the QSPR–GAP Model

To assess how well a model generalizes to new (or unseen) data, it is essential to perform an external validation. Often, external validation is performed on a reserved test set used only for this purpose, while model selection and/or tuning is performed on the training set (the remaining part of the data set) during an internal validation.^20,21,32,54^ A drawback in selecting a dedicated test set is possible selection bias, i.e., bias due to random fluctuations in smaller data sets. To avoid this, we iteratively select different test sets such that all of the data points are eventually used in a test set. Full details of the external and internal validations are outlined in SI Sec. S–III.

Briefly, the external validation was performed using a repeated five-fold cross validation (five-fold CV), where the full data set was shuffled randomly and subsequently partitioned into five distinct subsets. A test set was iteratively selected from the five subsets, and in each iteration, the remaining four subsets were combined into a single training set. Internal validation was performed on the training set at each iteration to tune the model “hyperparameters” (e.g., the number of principal components in PCR and PLS or the degree of shrinkage in ridge and lasso; see SI Sec. S–II). This procedure was repeated 10 times, leading to 50 different training–test splits, each with a unique combination of polymers. One important aim of this procedure is to ensure that many test sets contain polymers with fragment IDs absent from the training set, which enables efficient probing of the robustness of our proposed QSPR–GAP approach. Such out-of-training set fragment occurrences, in the following referred to as “out-of-sample fragments”, were identified 34 times for the 50 different training–test splits (Figure S14, SI).

Our proposed QSPR–GAP method is benchmarked against the pure GAP and QSPR methods. As mentioned above, the GAP models were fit using the OLS; however, the coefficients corresponding to the out-of-sample fragments were modified *a posteriori* to represent the mean of the coefficients associated with the in-sample fragments. This ad-hoc modification was performed to improve the robustness of the GAP model’s out-of-sample fragment coefficient estimates, which would otherwise be zero based on the least-squares fit. Again, the need for this ad-hoc approach highlights the fundamental problem with the GAP method.

Figure 2a–c displays the predicted *T̂*_g_ versus experimental *T*_g_ for a representative training–test split across the three different methods as representative examples. In these figures, blue circles represent the training set data, while orange stars indicate the test set data. We include results for the QSPR lasso model in panel (a), the QSPR–GAP lasso model in panel (b), and the GAP *L–Ar–L* model in panel (c). For this particular partition (training–test split) of the data, fragments with *i* = 8 and 30 do not exist in the training set. For the GAP model, these two out-of-sample fragments manifest as three clear outlier polymers, which the model can not handle (outlined orange stars in Figure 2a–c). Since these fragments are absent in the training set, the corresponding β̂_*i*_ values are not known, and we thus set β̂_8_ and β̂_30_ to the means of the in-sample fragment contributions, leading to the outlier polymers.

**Figure 2:**
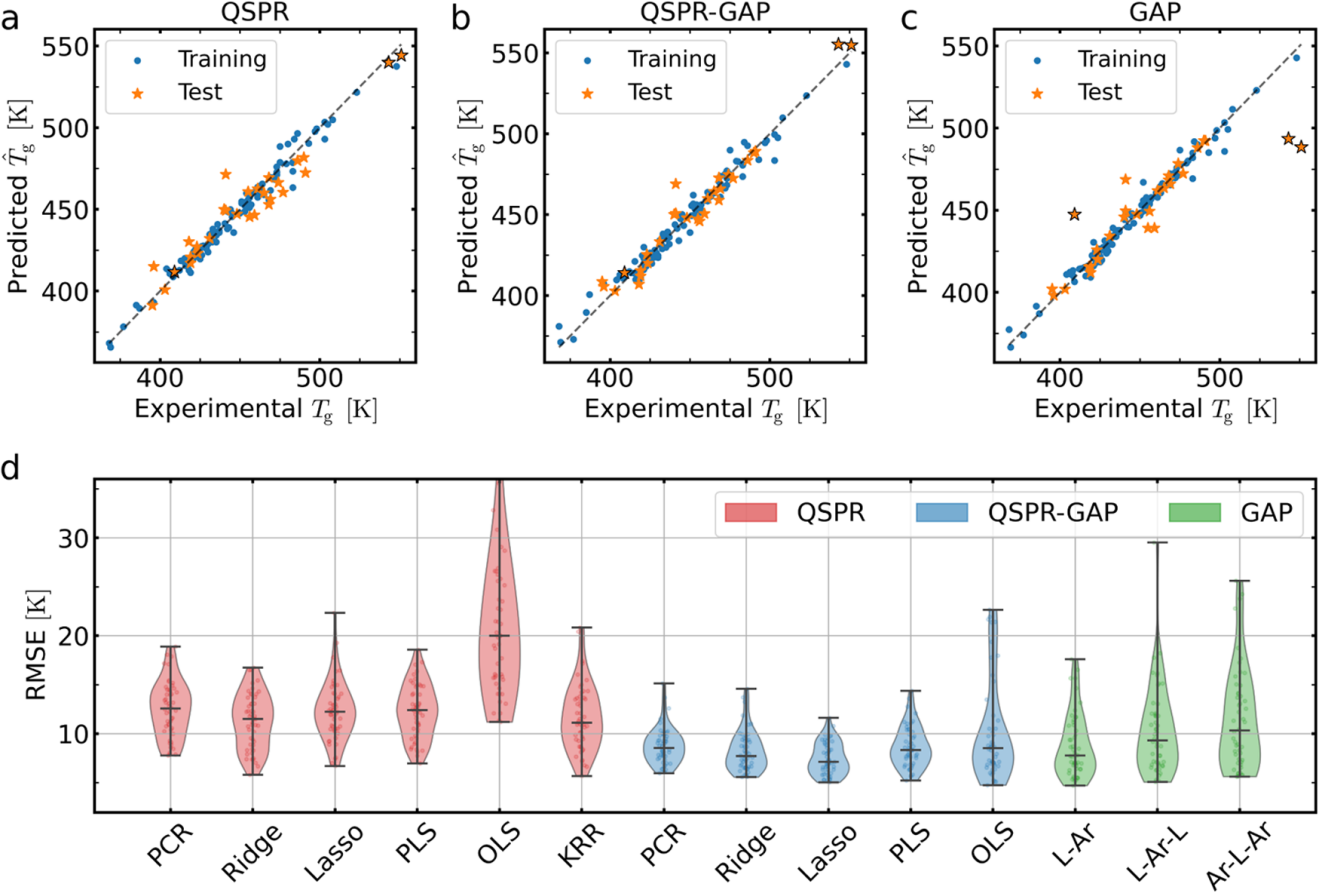
Benchmarking the QSPR–GAP model. (a–c) Comparing QSPR, QSPR–GAP, and GAP models from a single training–test split, where four polymers (indicated by the black marker edge color) in the test set contain at least one out-of-sample (*L*–Ar–*L*) fragment: (a) QSPR lasso model, (b) QSPR–GAP lasso model, and (c) GAP *L*–Ar–*L* model. (d) All models are compared by the distributions of their root mean square error (RMSE) during external validation, which involves predictions of the test set for 50 different training–test splits. RMSE distributions are represented using violin plots, where the outer envelope represents a kernel density estimation of the data (which is faintly displayed as points within the envelope). The black centerline represents the median, while the ends mark the extrema of the data. We note that the full distribution for the QSPR OLS goes beyond the scale and is truncated for a clearer comparison between the other models—its upper extrema extends as high as 59 K.

The advantages of the QSPR–GAP and QSPR approaches become obvious when results from the same data partitions involve the same out-of-sample fragments. The QSPR (lasso) model in Figure 2a and QSPR–GAP (lasso) model in Figure 2b demonstrate a significantly more robust prediction than the GAP model for the three outlier polymers. The root mean square error (RMSE) from the full external validation, i.e., the results from the full 50 training–test splits, is presented in Figure 2d for all investigated QSPR, QSPR–GAP, and GAP models. For the GAP model, in addition to the *L–Ar–L* fragment definition, we also investigated the definitions of *L–Ar* and *Ar–L–Ar*. From 50 splits, out-of-sample fragments are found 30 times for *L–Ar*, 34 times for *L–Ar–L*, and 40 times for the *Ar–L–Ar* fragment choice. The increase in the number of out-of-sample fragments grows with the number of available combinations of *L* and *Ar* groups (Figure 1A).

As shown in Figure 2d, even though all investigated QSPR–GAP models perform similarly (apart from OLS, as expected), the lasso model is the most accurate, with a RMSE range of ≃5–12K (depending on the partitioning of training/test data) and a median RMSE of ≃8 K. The QSPR models generally show a weaker predictive performance compared to QSPR–GAP, with median RMSE values ranging between 11 and 13 K (excluding OLS), compared to 8–9 K for QSPR–GAP. Thus, the QSPR–GAP models (PCR, ridge, PLS, and lasso) are more robust against the outlier (out-of-sample fragment-containing) polymers than the GAP models, and improve the predictive performance compared to the QSPR models, as shown in Figure 2.

Since the predictive ability (characterized by the RMSE) for the GAP models is significantly affected by the outlier polymers caused by out-of-sample fragments, we also compared models for which all outliers were removed (Figure S15, SI). We find that the predictions of the QSPR–GAP models are slightly improved, as exemplified by an RMSE ≃5–9 K for the lasso method, whereas the GAP models demonstrate a highly improved RMSE of ≃5–8 K. Interestingly, when comparing the predictive performance with out-of-sample fragment occurrences removed, the QSPR–GAP OLS results are identical to those of the GAP *L–Ar–L* model (Figure S15, SI).

Overall, QSPR–GAP leverages the strengths of QSPR with respect to its robustness against out-of-sample fragment-containing polymers while achieving comparable accuracy to GAP for polymers comprising only in-sample fragments. The GAP model shows excellent predictive performance for polymers containing fragments that are well represented by the data, with the downfall that polymers comprising out-of-sample fragments cannot conventionally be predicted. Although the method of averaging in-sample fragment contributions offers a solution, as demonstrated for GAP, a more accurate approach is to use descriptors to inform these contributions, which is what QSPR–GAP does.

When the QSPR method is applied (to an entire monomer), the descriptors implicitly encode conformational information, which appears to decrease predictive accuracy for *T*_g_. In contrast, when the descriptor calculations are focused on submonomer fragments (characterized by minimal conformational degrees of freedom), as in QSPR–GAP, the predictive accuracy improves. Thus, for QSPR, 3D descriptor encoding of conformational information for the isolated monomeric unit may introduce more error than insight, whereas the most critical information for *T*_g_ resides at the local intrafragment scale (with little or no conformational degrees of freedom).

## Descriptor Analysis

### Identifying the Most Important Descriptors

The key feature of the genetic algorithm models (QSPR–GAP GA*m*_GA_) is that they explicitly select a subset of (*m*_GA_ = 1, ..., 10) descriptors that best predict *T*_g_. This allows analysis of the direct impact of a discrete set of optimized descriptors on *T*_g_, which may help develop an understanding of their physical significance.

In testing the performance of the GA models, the same external validation consisting of a five-fold CV (repeated 10 times) is used, resulting in a total of 50 independent out-of-sample predictions, i.e., 50 different training–test splits (SI, Sec. S–III). The RMSE results for the 10 investigated QSPR–GAP GA*m*_GA_ models are shown in Figure 3a. Remarkably, only a few descriptors are needed for a good prediction. The most significant improvement in the predictive accuracy occurs between *m*_GA_ = 1 and 2, while for *m*_GA_ > 2, there is no significant improvement. Hence, only two descriptors are necessary (for this data sample) to predict *T*_g_ with an RMSE of ≃6–15 K. Thus, in the following, we restrict our attention to the two-descriptor model, QSPR–GAP GA2.

**Figure 3:**
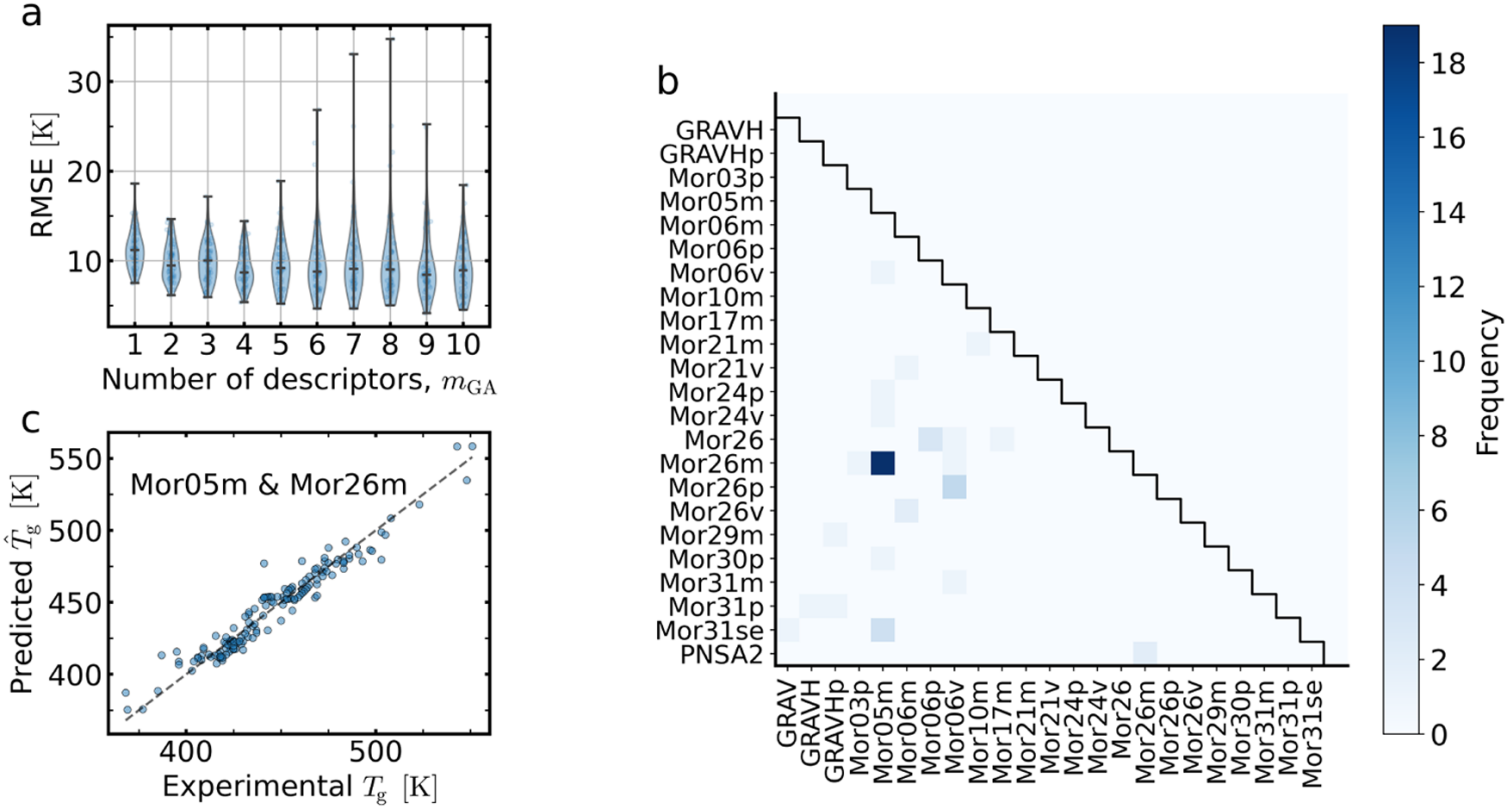
Analysis of descriptors. (a) Distributions (kernel density estimation) of the root mean square error (RMSE) for ten genetic algorithm (GA) models, consisting of a GA-based selection of *m*_GA_ = 1, *m*_GA_ = 2, ... *m*_GA_ = 10 optimal descriptors (from a pool of 213), followed by OLS regression. The RMSE is determined from a fivefold cross validation repeated ten times (while randomly shuffling each time), resulting in 50 distinct training–test splits. (b) GA results for *m*_GA_ = 2: from the complete set of two-descriptor combinations, we show all pairs selected at least once. As shown, the descriptor pair Mor05m and Mor26m was selected 19 times for the 50 training–test splits. (c) Predicted (in-sample) vs actual *T*_g_ values from an OLS regression on the full data set based only on the two descriptors Mor05m and Mor26m.

Figure 3b illustrates the frequency by which the GA selected different pairs of descriptors. It is clear that one pair stands out: Mor05m and Mor26m. From the 22,578 possible pairs arising from 213 descriptors, this pair was selected 19 out of 50 times (Figure 3b) from the 50 random data partitions. Descriptors Mor05m and Mor26m belong to the 3D-MoRSE family (Molecular Representation of Structures based on Electronic diffraction),^48–50^ which describes the 3D structure of a given fragment (or molecule) by a “form factor” based on atom-to-atom pair distances

4 $$I\left(q\right)=\sum_{l=1}^{N-1}\sum_{k=l+1}^{N}A_{k}A_{l}\frac{\sin\left(qr_{\mathrm{kl}}\right)}{qr_{\mathrm{kl}}}$$

where *k* and *l* label specific atoms, *N* is the number of atoms in the fragment, *A*_*k*_ and *A*_*l*_ are weighting factors for atoms *k* and *l*, *q* is the “scattering” wave vector, and *r*_*kl*_ is the Euclidean distance between atoms *k* and *l*.

Our descriptor set (*m* = 213 descriptors calculated using Mordred) contains 160 3D-MoRSE descriptors, characterized by 32 different *q* values: 0 (Mor01), 1 Å^–1^ (Mor02), 2 Å^–1^ (Mor03), ..., 31 Å^–1^ (Mor32) and five different weighting schemes for *A*_*k*_ and *A*_*l*_: unweighted (*A*_*k*_ = *A*_*l*_ = 1), atomic mass (MorXXm), van der Waals atomic volume (MorXXv), Sanderson electronegativity (MorXXse),^55^ and polarizability (MorXXp); all weighting schemes are scaled by their value for carbon. MoRSE descriptors Mor05m and Mor26m correspond to *q* = 4 Å^–1^ and *q* = 25 Å^–1^, where *A*_*i*_ is the ratio of the mass of atom *i* to the mass of carbon.

The *T*_g_ contribution β̂_*i*_ for fragment *i* can thus be accurately estimated by

5 $${\mathrm{\beta ̂}}_{i}={\mathrm{\gamma ̂}}_{0}+{\mathrm{\gamma ̂}}_{1}I_{i}\left(4\right)+{\mathrm{\gamma ̂}}_{2}I_{i}\left(25\right)$$

where *I*_*i*_(4) and *I*_*i*_(25) are calculated from the set of atoms in fragment *i* and γ̂_μ=0,1,2_ are the regression coefficients, estimated with an OLS regression applied to the full data sample of 146 polymers (no training–test splits); these estimates are provided in Table 1. The predicted against experimental *T*_g_ results are shown in Figure 3c, with an in-sample RMSE of ≃8 K.

**Table 1:** Regression Coefficients from the Best Two-Descriptor Genetic Algorithm Model, as Estimated from the Full Data Set of 146 Polymers (OLS)^a^

μ	descriptor	γ̂_μ_ [K]	CI (95%) L/U [K]
0		298	286/310
1	Mor05m	–58	–67/–50
2	Mor26m	–198	–239/–157

a Upper and lower 95% confidence intervals (CIs) are presented for the estimated coefficients *γ*_0_ and *γ*_μ_. For assumptions and diagnostics of distributions, see the SI, Sec. S–IV.

### Atomic-Level *T*_g_ Contributions

Using eqs 4 and 5, we can express the estimated *T*_g_ contribution of fragment *i* as a sum over atomic pair contributions

6 $${\mathrm{\beta ̂}}_{i}={\mathrm{\gamma ̂}}_{0}+\sum_{l=1}^{N_{i}-1}\sum_{k=l+1}^{N_{i}}{\mathrm{\pi ̂}}_{\mathrm{kl}}$$

where π̂_*kl*_ denotes the (estimated) *T*_g_ contribution given by the pair of atoms *k* and *l*, expressed in terms of the two descriptors Mor05m and Mor26m,

7 $${\mathrm{\pi ̂}}_{\mathrm{kl}}={\mathrm{\gamma ̂}}_{1}M_{k}M_{l}\frac{\sin\left(4r_{\mathrm{kl}}\right)}{4r_{\mathrm{kl}}}+{\mathrm{\gamma ̂}}_{2}M_{k}M_{l}\frac{\sin\left(25r_{\mathrm{kl}}\right)}{25r_{\mathrm{kl}}}$$

*M*_*k*_ is the mass of atom *k* (divided by the mass of carbon), and *r*_*kl*_ is the distance between atom pair *k*,*l*. To determine the fragment contribution β̂_*i*_ , the π̂_*kl*_ contributions are summed over the total number of atoms *N*_*i*_ in the *i*th fragment according to eq 6.

Function π̂_*kl*_ (*r*_*kl*_ , *M*_*k*_ *M*_*l*_) is shown in Figure 4a, and at constant *r*_*kl*_ , π̂_*kl*_ is a linear function of the product *M*_*k*_M_*l*_ of atomic masses, as illustrated by the color gradient, and at constant *M*_*k*_M_*l*_ , π̂_*kl*_ is an oscillating function of the pair distance. As *r*_*kl*_ increases, the pair contributions π̂_*kl*_ become less relevant to the overall β̂_*i*_ , with negative and positive contributions canceling out in the summation of eq 6. Thus, the most important contributions lie within the range *r*_*kl*_ ≃ 1.2–1.5Å.

**Figure 4:**
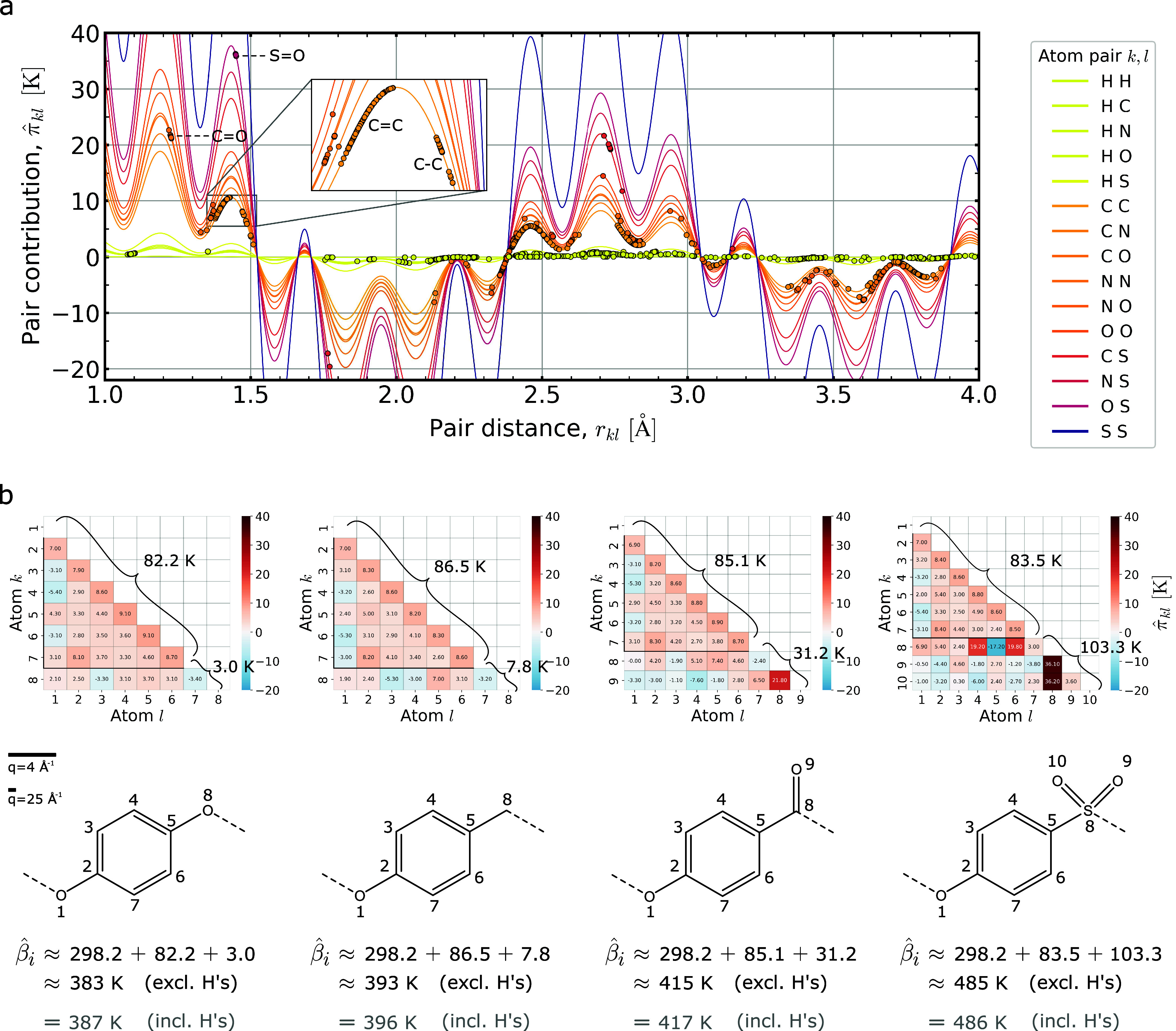
Estimated interatomic pair *T*_g_ contributions. (a) Estimated function π̂_*kl*_ of pairwise atomic *T*_g_ contributions, as expressed in eq 7; π̂_*kl*_ is a function of the pair distance *r*_*kl*_ between *k* and *l* atoms and the product *M*_*k*_M_*l*_ , where the latter is illustrated by the color gradient. The lines represent the estimated function π̂_*kl*_(*r*_*kl*_ , *M*_*k*_ *M*_*l*_), and the solid points represent the function evaluated for the specific atom pairs within every polymer in the data set. The inset is a magnification of the range containing contributions from different carbon–carbon single and double bonds, whose bond lengths (when energy-minimized) vary slightly depending on the specific fragment. (b) Four example fragment IDs that share the same structure (in atoms 1–7) but vary by a single functional group (in atoms 8+). The pairwise contributions π̂_*kl*_ are shown as colored tiles with their values shown inside each tile. The scale bars show the length scales (1.6 and 0.3Å) corresponding to *q* = 4 Å^–1^, 25 Å^–1^, relative to the size of the (planar) structures. In the sum over pair contributions (eq 6) atoms *l* = 1, ..., 6 and *k* = 2, ..., 7, where *l* < *k* (indicated by the partition) correspond to the same structure and yield nearly the same contribution Δ*β̂*_*i*_ ≃ 82.2–85.5 K for all four fragments. The remaining atom pairs set the fragments apart in their summed contribution to β̂_*i*_, which varies from Δ*β̂*_*i*_ ≃ 3.0 to 103.3 K. Atomic pair contributions including hydrogen atoms have been ignored from the plot since they show only small contributions to β̂_*i*_ (according to eq 7).

In Figure 4b, we show four *L*_1_–Ar–L_2_ fragments for which only the linker *L*_2_ differs. As discussed above, the structure of each fragment is energy-minimized (using MMFF) and is represented by a unique set of *r*_*kl*_ , *M*_*k*_ , and *M*_*l*_ values. For each of the four fragments, the function π̂_*kl*_(*r*_*kl*_ , *M*_*k*_ *M*_*l*_) is evaluated for all atomic pairs, and the results are provided in the tiles (also illustrated by the corresponding heat map); each tile represents the pairwise atomic *T*_g_ contribution from atoms *k* and *l*. The overall fragment *T*_g_ contribution β̂_*i*_ results from a sum over all atomic pair contributions π̂_*kl*_ and the constant γ̂_0_.

Since hydrogen-containing pairs show very small *T*_g_ contributions (as shown in Figure 4a), they are omitted for clarity in Figure 4b. The contributions from atoms 1–7 are very similar because these atoms represent the same molecular structure motif (ether-linked phenyl). Hence, the sum over π̂_*kl*_ for atoms *k*,*l* = 1, ..., 7 gives contributions of Δ*β̂*_*i*_ = 82.2, 86.5, 85.1, and 83.5 K, respectively, for the four fragments, as shown in Figure 4b. The slight differences between these values arise when minimizing the energy because the interatomic distances *r*_*kl*_ are influenced by the atoms in linker *L*_2_. The differences in linker *L*_2_ (atoms 8+) lead to significant differences in *T*_g_, with contributions of Δ*β̂*_*i*_ = 3.0, 7.8, 31.2, and 103.2 K for the four structures. The total *T*_g_ contributions for the four fragments are β̂_*i*_ = 383, 393, 415, and 485 K, respectively, determined using eq 6 while excluding hydrogens from the sum.

We conclude that for PAEK polymers the *T*_g_ contribution of each fragment (β_*i*_) is very well approximated as a constant plus a sum over all atomic pair contributions (π̂_*kl*_), where the main contributions correspond to short atomic pair distances of 1.2–1.5 Å. The genetic algorithm identified two important 3D-MoRSE descriptors corresponding to length scales (∼2π/*q*) of 1.6 and 0.3 Å (see the scale bars in Figure 4b). The former is approximately the size of the average single C–C bond in the data set (Figure 4a), whereas the latter corresponds to a length scale much smaller than an interatomic bond. Physically, the reliability of MMFF would be lost at a length scale of 0.3 Å; however, based on the figure, it is evident that it still encodes the length differences between the types of bonds. For example, in the zoomed inset of Figure 4a, the higher-frequency *q* component in the function induces a modulation that distinguishes between the C–C and C = C bonds. Without the length scale at *q* = 25Å^–1^, the fit would not have enough flexibility to appropriately capture the differences among the S = O, C = O, C = C, C–C, ... (as labeled in the figure). We note that regularized sparse linear regression methods (such as lasso) applied to all 32 mass-weighted 3D-MoRSE descriptors likely yield a model with improved predictive accuracy and finer differentiation between bond types.

The example shown in Figure 4b indicates that the linker properties, such as their bulkiness, have the greatest impact on *T*_g_; see, for example, the high contribution from S = O (36 K) and C = O (22 K) in Figure 4a,b. Based on these findings, we speculate that these particular groups strongly restrict torsional rotations and increase the chain’s stiffness, thus raising *T*_g_. For the glass transition in general, the interplay between packing and chain flexibility^5,16^ suggests that three-body features, such as intramolecular angles, play an important role. In this study, such features are likely implicitly incorporated due to the chosen *L–Ar–L* motif.

## Conclusions

We present a new method for predicting *T*_g_ from the monomer structure in polymers. The method combines group additive properties (GAP) with a quantitative structure–property relationship (QSPR) approach. The GAP method assumes that *T*_g_ can be expressed by a molar mass-weighted average over *T*_g_ contributions from submonomer motifs (fragments), and our QSPR–GAP model uses molecular descriptors to relate the physical properties of these fragments to their GAP-like *T*_g_ contributions. We apply this model to a data set of 146 linear poly­(aryl ether ketone) (PAEK) homo- and copolymers, resulting in a median root mean square error of 8 K (out-of-sample).

Compared to the standalone GAP and QSPR methods, the QSPR–GAP method improves robustness and accuracy in out-of-sample *T*_g_ predictions. Furthermore, 3D descriptor calculations for submonomer fragments are significantly faster than in traditional QSPR approaches, which are based on monomers (or oligomers), due to the reduction in conformational degrees of freedom.

Using a genetic algorithm, we show that only two molecular descriptors (from a pool of 213) are necessary to predict *T*_g_ with an RMSE of ≃6–15 K. Moreover, we identify a direct mapping between *T*_g_ and the monomer structure through pairwise atomic contributions.

This work offers an accurate, accessible, and broadly applicable predictive model suitable for small data sets and deployment on a standard laptop. The QSPR–GAP method is transferable to other classes of polymers, both synthetic and natural (e.g., conjugated or biopolymers), and to physical behavior beyond the glass transition, such as mechanical, optical, or transport properties.

## Methods

The number of occurrences of fragment *i* in polymer *a* is

8 $${\left(X\right)}_{\mathrm{ai}}\equiv X_{\mathrm{ai}}$$

where **X** is an *n* × *p* dimensional count matrix, with *n* rows representing the full set of polymer IDs and *p* columns representing the full set of unique fragment IDs. We normalize **X** by the molar mass of the repeating unit, resulting in the mass-weighted composition matrix

$$\mathrm{X̅}={\left(\mathrm{diag}\left[XM\right]\right)}^{-1}X\left(\mathrm{diag}\left[M\right]\right)$$ 9

where $M\in R^{p}$ is a *p*-vector that enumerates the fragment molar masses. Note that the molar mass *M*_*i*_ of an *L–Ar–L* fragment is the molar mass of half of each *L* group and the full *Ar* group: *M*_*i*_ = *M*_*L*_*i*1__/2 + *M*_*Ar*_*i*__ + *M*_*L*_*i*2__/2. Since the same *L* groups are counted twice when building a repeat unit structure from a given set of fragment IDs, the product **X*M*** encompasses the molar mass of the repeating unit for all polymers in the data set (or correspondingly the molar mass of a copolymer’s repeating unit, averaged over its comonomer mass fractions).

### GAP Model

We used ordinary least-squares (OLS) to estimate the *p* coefficients β̂_*i*_ , i.e., the *p*-vector $\mathrm{\beta ̂}\in R^{p}$ , by minimizing the residual sum of squares

$$\mathrm{\beta ̂}=\underset{\beta }{\arg\min}\left\{\overset{n}{\underset{a=1}{\sum }}(T_{g}^{a}-f{\left({\left(\mathrm{X̅}\right)}_{a}\right))}^{2}\right\}$$ 10

where the linear fitting function *f* that approximates *T*_g_^*a*^ (of the *a*th polymer in the training data) is given by *f*((**X̅**)_*a*_) = ∑_*i*=1_^*p*^ X̅_*ai*_ β_*i*_ ≡ (**X̅** **β**)_*a*_ . The solution, i.e., the least-squares estimate of β, is given by

$$\mathrm{\beta ̂}={\left({\mathrm{X̅}}^{T}\mathrm{X̅}\right)}^{-1}{\mathrm{X̅}}^{T}T_{g}$$ 11

where the *n*-vector $T_{g}\in R^{n}$ contains all *n* $T_{g}^{a}$ values in the training sample. Note that each estimated coefficient β̂_*i*_ corresponds to the predicted glass transition temperature of a polymer solely comprising fragment *i* as its repeating monomer.

An out-of-sample prediction of the glass transition temperature for a polymer *b* with fragment composition *X̅*_*bi*_ (given all *i* = 1, ..., *p*) can now be determined as

12 $${\mathrm{T̂}}_{g}^{b}=\sum_{i=1}^{p}{\mathrm{X̅}}_{\mathrm{bi}}{\mathrm{\beta ̂}}_{i}$$

Predictions of *T*_g_^*b*^ are restricted to polymers consisting of fragments whose contributions β_*i*_ have already been estimated from eq 11, meaning that GAP predictions are chemically constrained to polymers consisting of fragments within the set {1, ..., *p*}.

### QSPR–GAP Model

The QSPR–GAP model contains a *descriptor matrix D*_*iμ*_ , which encodes the chemical and physical properties of the *i*th fragment ID in terms of μ = 1, ..., *m* descriptor values

13 $${\left(D\right)}_{i\mu }\equiv D_{i\mu }$$

We then express each fragment *T*_g_ contribution β_*i*_ as a linear combination of the *m* descriptors

14 $$\beta_{i}=\gamma_{0}+\sum_{\mu =1}^{m}D_{i\mu }\gamma_{\mu }$$

where the (*m*+1)-vector $\gamma \in R^{\left(m+1\right)}$ contains the regression coefficients. The zeroth column index is included in the matrix **D** as *D*_*i*0_ = 1 for all fragments *i* = 1, ..., *p* to accommodate the constant term γ_0_; therefore, $D\in R^{p\times \left(m+1\right)}$ .

The methods used to estimate **γ** include (1) principal component regression, (2) ridge regression, (3) lasso regression, and (4) partial least-squares regression and are discussed further in the SI, Sec. S–II. However, to illustrate the application of the QSPR–GAP model in its simplest form, we discuss the genetic algorithm (GA) model here.

The GA uses concepts analogous to evolution to select an optimal subset (*m*_GA_ ≤ 10) from a total of 213 potential descriptors. The descriptors chosen will have the greatest influence on *T*_g_ and, once selected, are included in the function

15a $$f\left({\left(\mathrm{X̅}\right)}_{a},D\right)=\sum_{i=1}^{p}{\mathrm{X̅}}_{\mathrm{ai}}\beta_{i}$$

15b $$=\;\gamma_{0}+\sum_{i=1}^{p}\sum_{\mu =1}^{m_{\mathrm{GA}}}{\mathrm{X̅}}_{\mathrm{ai}}D_{i\mu }\gamma_{\mu }$$

­(since ∑_*i*=1_^*p*^ *X̅*_*ai*_ = 1), followed by a least-squares minimization

16 $$\mathrm{\gamma ̂}=\underset{\gamma }{\arg\min}\left\{\sum_{a=1}^{n}(T_{g}^{a}-f{\left({\left(\mathrm{X̅}\right)}_{a},D\right))}^{2}\right\}$$

yielding the solution

17 $$\mathrm{\gamma ̂}={\left(D^{T}{\mathrm{X̅}}^{T}\mathrm{X̅}D\right)}^{-1}D^{T}{\mathrm{X̅}}^{T}T_{g}$$

Once the coefficients **γ** are estimated from the training data, the estimated *T*_g_ contribution of any new fragment *j* is given by

18 $${\mathrm{\beta ̂}}_{j}={\mathrm{\gamma ̂}}_{0}+\sum_{\mu =1}^{m_{\mathrm{GA}}}D_{j\mu }{\mathrm{\gamma ̂}}_{\mu }$$

which may include fragments that are not in the original data sample.

An out-of-sample predicted glass transition temperature for a new polymer *b* given fragment compositions *X̅*_*bj*_ (for all *j* = 1, ..., *q*) and molecular descriptors *D*_*jμ*_ (for all μ = 1, ..., *m*_GA_) is

19 $${\mathrm{T̂}}_{g}^{b}={\mathrm{\gamma ̂}}_{0}+\sum_{j=1}^{q}\sum_{\mu =1}^{m_{\mathrm{GA}}}{\mathrm{X̅}}_{\mathrm{bj}}D_{j\mu }{\mathrm{\gamma ̂}}_{\mu }$$

noting that if {1, ..., *p*} is the set of in-sample fragments, then {1, ..., *q*} is the set of in-sample and out-of-sample fragments, where {1, ..., *p*} ⊆ {1, ..., *q*}.

## Supplementary Material



## Data Availability

Source data files are available at the University of Leeds Data Repository at 10.5518/1596.

## Notes

Description of the data set; QSPR feature generation including the conformer generation and descriptor calculations; background information on the machine learning models and algorithms used; description of the genetic algorithm implementation, model evaluation strategy, and cross validation; all fragment-level *T*_g_ contributions; and a few examples of atomic-level *T*_g_ contributions (PDF)

## Notes

**Code Availability Statement**: The codes that support the findings of this study are available upon reasonable request from the corresponding author.
