# Supplementary material for: Polymer Informatics Method for Fast and Accurate Prediction of the Glass Transition Temperature from Chemical Structure
Source: Macromolecules. 2025 Jun 16;58(13):6407–17. doi: 10.1021/acs.macromol.5c00178 (PMC12257600; doi:10.1021/acs.macromol.5c00178)
Supplement: Supplementary file 1 [file ma5c00178_si_001.pdf]

Supplementary Information for:
Polymer Informatics Method for Fast and Accurate Prediction of the Glass Transition Temperature from Chemical Structure

Sebastian Brierley-Croft[1], Peter D. Olmsted[2], Peter J. Hine[1], Richard J. Mandle[1], Adam Chaplin[3], John Grasmeder[3], and Johan Mattsson[1*]

[1] *School of Physics and Astronomy, University of Leeds, Leeds LS2 9JT, United Kingdom*

[2] *Department of Physics and Institute for Soft Matter Synthesis and Metrology, Georgetown University, Washington DC, 20057 and*

[3] *Victrex PLC, Hillhouse International, Thornton Cleveleys, Lancashire FY5 4 QD, United Kingdom*

(Dated: June 12, 2025)

CONTENTS

S-I. Data 2
- A. The data set of glass transition temperatures 2
- B. 3D-descriptor calculation 2
- C. Model inputs 3

S-II. Models 5
- A. Standardising variables 6
- B. Shrinkage methods 6
- C. PCR and PLS dimension reduction methods 7
- D. Genetic algorithm 8

S-III. Model evaluation 10
- A. Statistical models 11
- B. Genetic algorithm models 13
- C. Summary of results 14

S-IV. Fragment contributions $\hat{\beta}_i$ 16

S-V. Atom pair contributions $\hat{\pi}_{ij}$ 19

S-VI. Fragment definitions 21

References 22

* k.j.l.mattsson@leeds.ac.uk

S-I. DATA

A. The data set of glass transition temperatures

The data set contains glass transition temperatures (T_g) for a total of 77 homopolymers and 69 copolymers collected from an extensive list of references, and from measurements performed by the research and development team at Victrex. The distribution of the T_g-values in the data set is shown in Fig. S1.

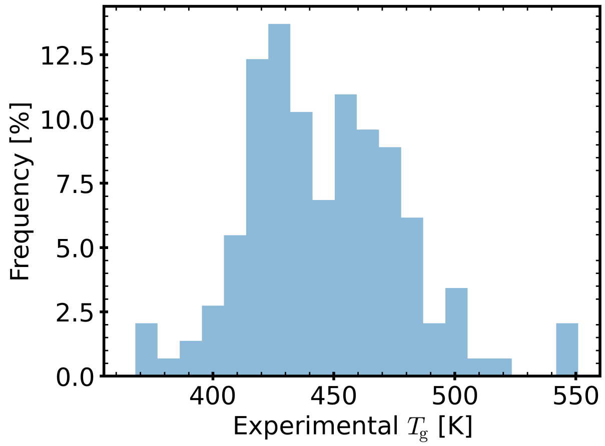

Figure S1: **Distribution of T_g values in the dataset.**

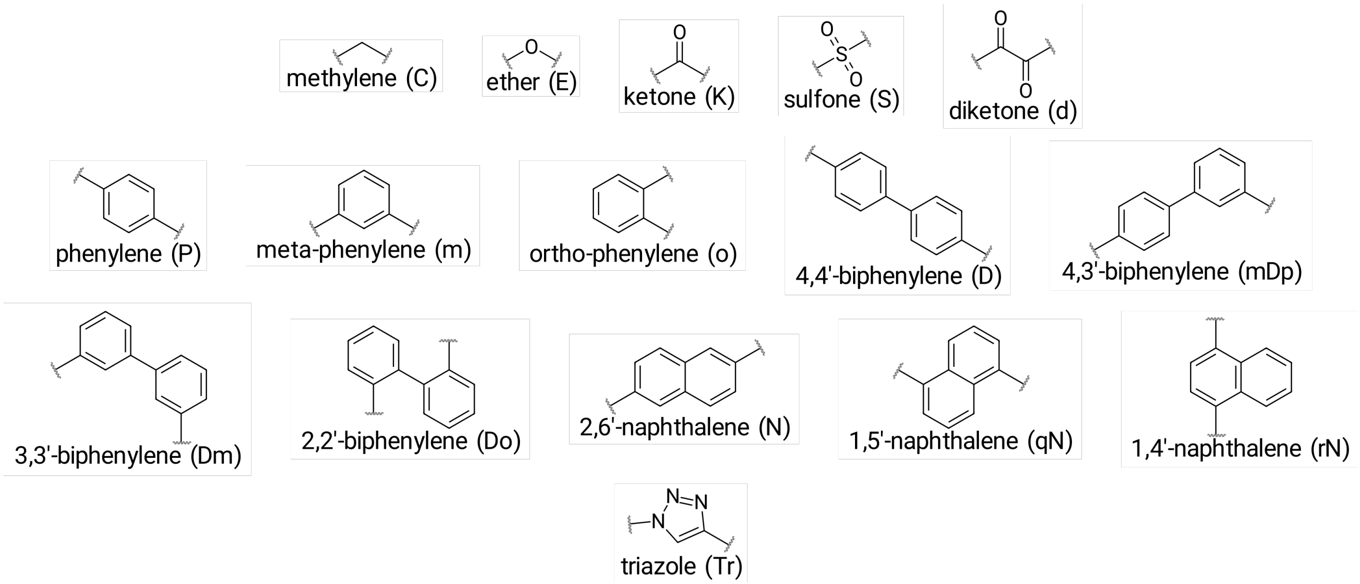

Figure S2: **Backbone Groups** that comprise the PAEK polymers in this data set. The structures on the first row are the linkers, while the remaining structures contain aryl groups. The structural abbreviations for each group are provided in brackets.

B. 3D-descriptor calculation

The process used to calculate the 213 3D-descriptors for the QSPR-GAP and QSPR methodsis as follows:

1. The set of motifs (*i.e.* the 30 *L-Ar-L* fragments or the 83 monomeric units) were represented as a simplified molecular-input line-entry system (SMILES) string [1, 2], with the dangling bonds end-capped with hydrogens.

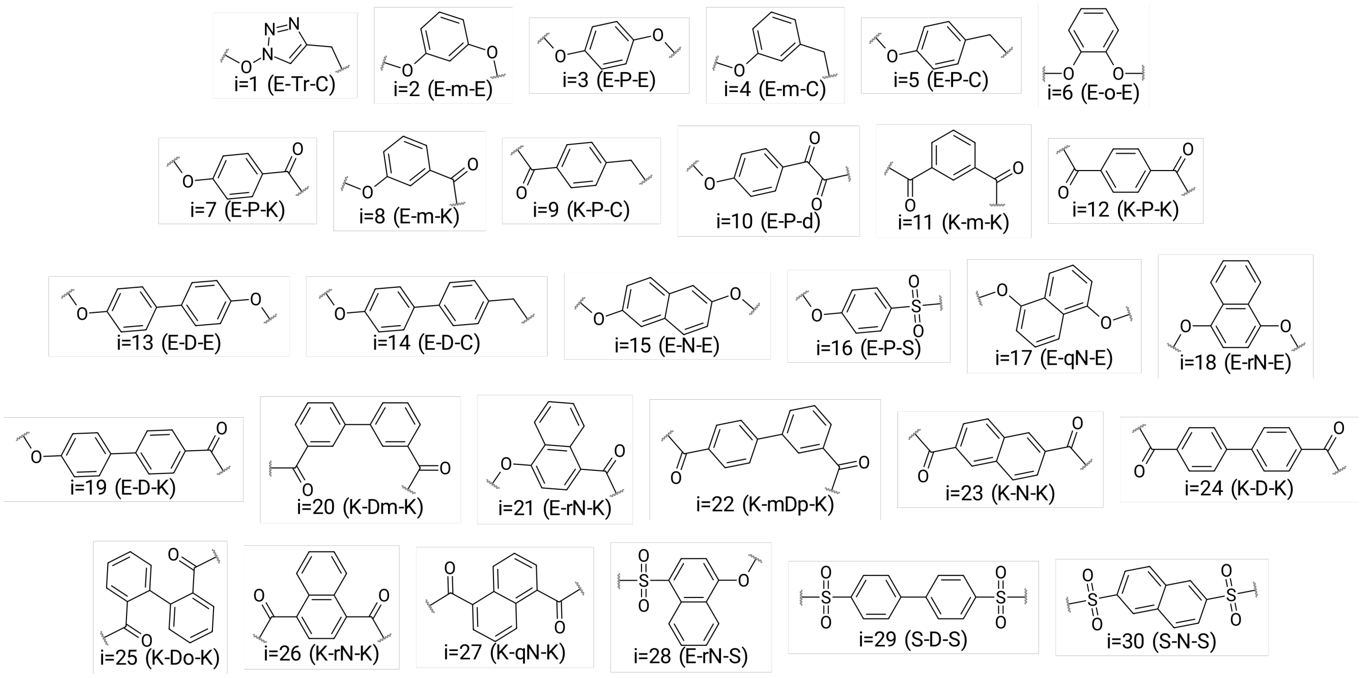

Figure S3: **All fragment structures** $i = 1, \ldots, 30$ present in the data set (L-Ar-L). The abbreviations of the functional groups are shown in brackets, following Fig. S2.

2. Each SMILES string was converted into a `rdkit.Chem.rdchem.Mol` object using RDKit [3], to create unoptimised 2D atomic coordinates for each motif.

3. A total of 500 initial 3D-conformations of each motif were generated using the RDKit function `rdkit.AllChem.EmbedMultipleConfs(mol, numConfs=500, params=AllChem.ETKDG())`.

4. The 3D-coordinates of the 500 conformations corresponding to each motif were energy-optimised based on the Merck Molecular Force Field (MMFF), using the RDKit function `rdkit.AllChem.MMFFOptimizeMoleculeConfs (mol, maxIters=1000)`. This resulted in a distribution of optimised conformer energies from which the lowest energy conformer was chosen for each motif.

5. `Mordred` [4] was used to calculate ($m = 213$) 3D-descriptors of the lowest energy conformer.

C. Model inputs

In the Group Additive Properties (GAP) approach the count matrix $\mathbf{X}$ is molar mass normalised giving the composition matrix $\bar{\mathbf{X}}$ with elements $\bar{X}_{ai}$. β_i is then estimated from the experimentally available T_g values by Ordinary Least Squares (OLS) regression against $\bar{\mathbf{X}}$. The column space of $\mathbf{X}$, and thus the elements X_{ai}, vary depending on the initial definition of a 'fragment'. For the GAP analysis, three different versions of $\mathbf{X}$ were investigated, corresponding to the fragment definitions: L-Ar, L-Ar-L, and Ar-L-Ar, see Fig. S20. Since each polymer is represented as a small subset of the full set of available fragments, the matrix $\mathbf{X}$ is sparse. To illustrate this, we present $\mathbf{X}$ and $\bar{\mathbf{X}}$ as a heat map in Fig. S4 (using the L-Ar-L definition); it is clear from the heat map that the ether-phenyl-ketone (E-P-K) fragment ($i = 7$) contributes significantly to the polymer composition throughout the data set. In this work, we have focused on only fragment definitions that join the L and Ar groups in the fragment identities. Defining count matrices with L and Ar groups represented separately in the columns of $\mathbf{X}$ should be approached with caution. This is because the sequence of alternating Ar and L groups, in the general PAEK monomer structure, leads to the linear constraint that the number of Ar groups is equal to the number of L groups for every polymer in our data set. This linear dependence leads to a non-invertible matrix $\bar{\mathbf{X}}^\top \bar{\mathbf{X}}$, which causes issues for the GAP estimation in Eq. (11) in the main text.

The QSPR-GAP method combines the assumptions of GAP, with quantitative structural properties (QSPR) calculations, requiring the matrix $\bar{\mathbf{X}}$ to quantify the fragment composition in a given monomer and a descriptor matrix

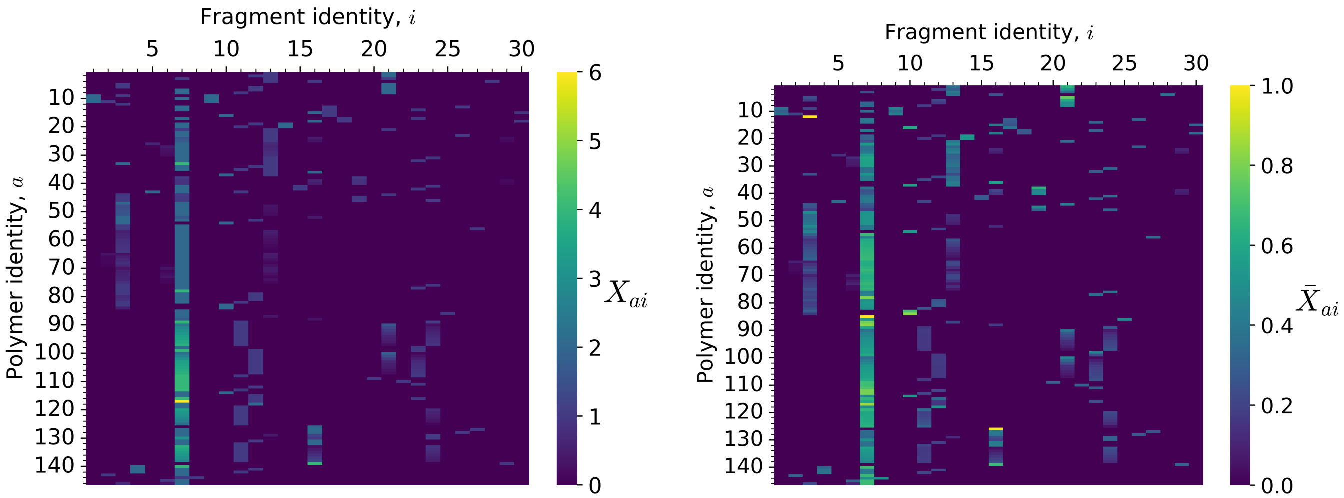

(a) $\mathbf{X}$

(b) $\bar{\mathbf{X}}$

Figure S4: **Fragment count and composition matrices.** Heat maps of **(a)** the fragment count matrix $\mathbf{X}$ and **(b)** the molar mass-weighted fragment composition matrix $\bar{\mathbf{X}}$ calculated from $\mathbf{X}$. Plots indicate the degree of sparsity in the matrices, as well as the number and content of fragments per polymer identity. The fragment identity i corresponds to Fig. S3.

$\mathbf{D}$ to encode the physical properties of the constituent fragments. The information contained in the descriptor matrix $\mathbf{D}$ can be presented in terms of the Pearson correlation matrices $\mathcal{P}_{\mu\nu}^{(\mathbf{D},\mathbf{R})}$ for $\mathbf{D}$ and the matrix product $\mathbf{R} \equiv \bar{\mathbf{X}}\mathbf{D}$. The Pearson correlation matrix quantifies how different descriptors μ and ν are correlated with each other either across the fragments in the data set ($\mathcal{P}_{\mu\nu}^{(\mathbf{D})}$) or across the set of polymers ($\mathcal{P}_{\mu\nu}^{(\mathbf{R})}$). Hence,

$$\mathcal{P}_{\mu\nu}^{(\mathbf{D})} = \frac{\sum_{i=1}^{p}(D_{i\mu} - \overline{D}_{\mu})(D_{i\nu} - \overline{D}_{\nu})}{\sqrt{\sum_{i=1}^{p}(D_{i\mu} - \overline{D}_{\mu})^2}\sqrt{\sum_{i=1}^{p}(D_{i\nu} - \overline{D}_{\nu})^2}}, \tag{S1}$$

where $\overline{D}_{\mu} \equiv \frac{1}{p}\sum_{i=1}^{p} D_{i\mu}$ is the mean of descriptor μ over all fragments $i = 1, \ldots, p$. Similarly,

$$\mathcal{P}_{\mu\nu}^{(\mathbf{R})} = \frac{\sum_{a=1}^{n}(R_{a\mu} - \overline{R}_{\mu})(R_{a\nu} - \overline{R}_{\nu})}{\sqrt{\sum_{a=1}^{n}(R_{a\mu} - \overline{R}_{\mu})^2}\sqrt{\sum_{a=1}^{n}(R_{a\nu} - \overline{R}_{\nu})^2}}, \tag{S2}$$

where $\overline{R}_{\mu} \equiv \frac{1}{n}\sum_{a=1}^{n} R_{a\mu}$ is the mean of descriptor μ over all polymers $a = 1, \ldots, n$.

The two Pearson correlation matrices, shown in Fig. S5, are very similar. Each correlation matrix has multiple regions of high correlations in the off diagonal elements, and in particular, collections of 3D-MoRSE descriptors (`Mor01`-`Mor32p`) exhibit strong correlations for the same q values but different weighting schemes. This is no surprise, since the included weighting scheme (atomic mass, van der Waals volume, electronegativity and polarisability) are strongly correlated on physical grounds. Hence we expect that many fewer descriptors will eventually suffice to capture the behavior of T_g.

A principal component analysis (PCA) was performed on the two matrices $\mathbf{D}$ and $\mathbf{R} \equiv \bar{\mathbf{X}}\mathbf{D}$. First, the matrices are standardised to obtain $\mathbf{D}^*$ and $\mathbf{R}^*$ (see Sec. S-II A) and then the eigenvalues of $\mathbf{D}^{*\top}\mathbf{D}^*/(p-1)$ and $\mathbf{R}^{*\top}\mathbf{R}^*/(n-1)$ are computed. Each eigenvalue of $\mathbf{D}^{*\top}\mathbf{D}^*/(p-1)$ constitutes the variance of the descriptors corresponding to a particular principle component, over the complete set of fragments. Hence, the total variance within the set of fragments is given by the sum of the eigenvalues. The eigenvalues of $\mathbf{R}^{*\top}\mathbf{R}^*/(n-1)$ similarly represent the variance of the mass-weighted descriptors within respective principal components, across the entire set of polymers. The relative variance captured by each eigenvalue is shown in the *scree plots* (Fig. S6), in which the eigenvalues, normalised by the sum of all eigenvalues, are plotted against the principal component (or eigenvalue) number in order of decreasing magnitude. This is referred to as *explained variance*. The figures show that more than 99% of the variance is captured by the first 15 principal components, despite there being $m = 213$ descriptors.

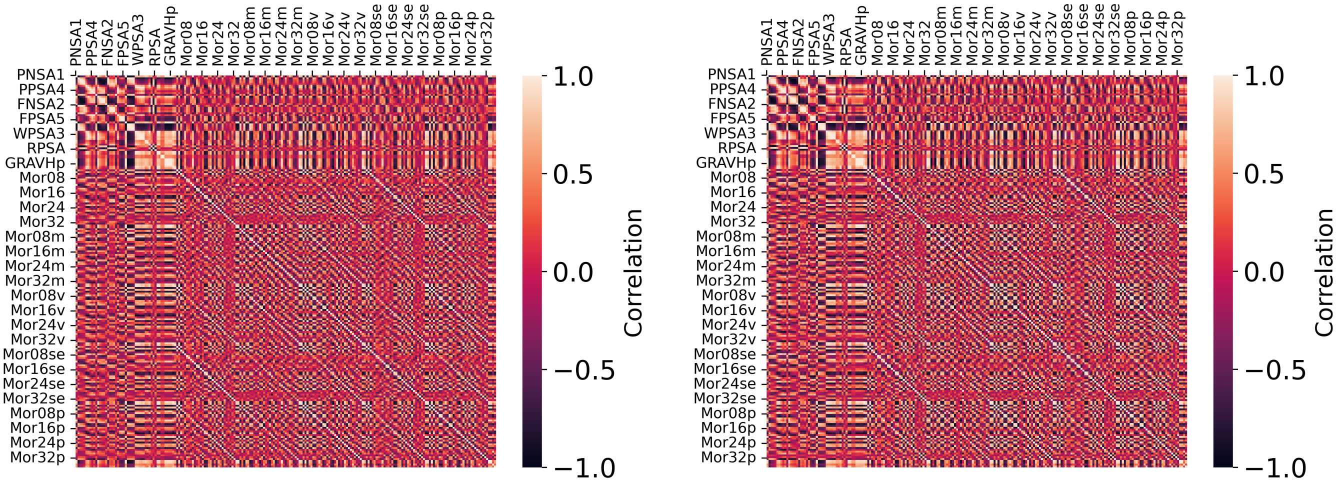

(a) Pearson Correlation matrix of $\mathbf{D}$.

(b) Pearson Correlation matrix of $\bar{\mathbf{X}}\mathbf{D}$

Figure S5: **Pearson Correlation Matrices** $\mathcal{P}_{\mu\nu}$ of **(a)** descriptor matrix $D_{i\mu}$ for all descriptors $\mu = 1, \ldots, 213$ and summed over the fragments $i = 1, \ldots, 30$ **(b)** $R_{a\mu} \equiv (\bar{\mathbf{X}}\mathbf{D})_{a\mu}$ for all descriptors $\mu = 1, \ldots, 213$ and summed over all polymers $a = 1, \ldots, 146$. The axis labels are displayed at every eighth descriptor, starting from the first, for example, $\mu = 1, 9, 17, 25, \ldots$ and so on.

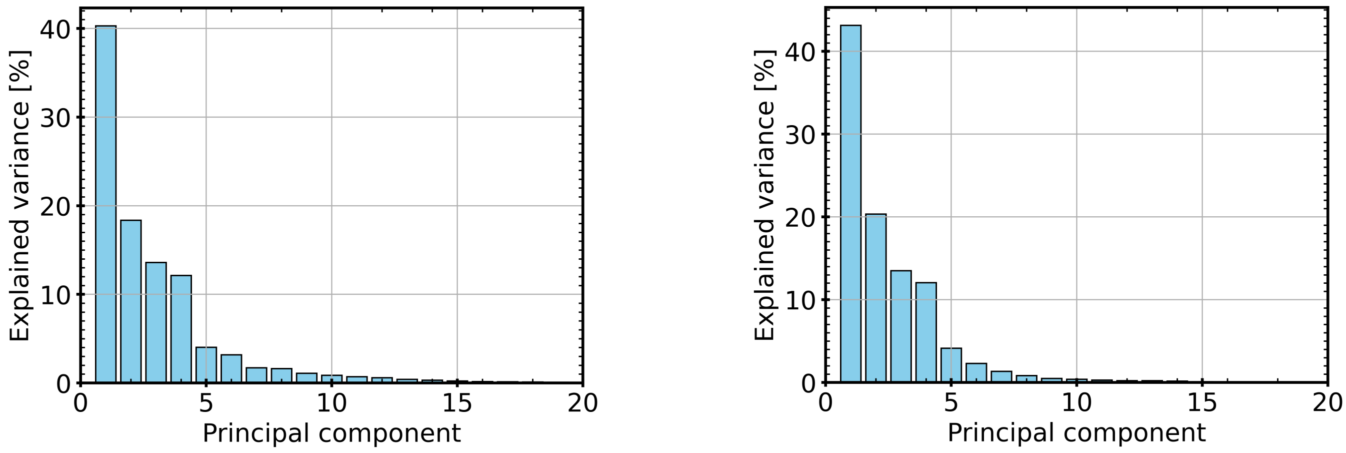

(a) Scree plot of $\mathbf{D}$

(b) Scree plot of $\bar{\mathbf{X}}\mathbf{D}$

Figure S6: **Principal component analysis of D and R** $\equiv$ $\bar{\mathbf{X}}\mathbf{D}$**.** The *scree plots* show the eigenvalues of $\mathbf{D}^{*\top}\mathbf{D}^{*}/(p-1)$ and $\mathbf{R}^{*\top}\mathbf{R}^{*}/(n-1)$. The *explained variance* is the ratio between an eigenvalue and the sum of all eigenvalues.

S-II. MODELS

For the QSPR-GAP method, the ordinary least squares (OLS) method is not appropriate for the estimation of the coefficients γ_0 and γ_μ ($\mu = 1, \ldots, m$) in the fitting function

$$f((\bar{\mathbf{X}})_a, \mathbf{D}) = \gamma_0 + \sum_{i=1}^{p} \sum_{\mu=1}^{m} \bar{X}_{ai} D_{i\mu} \gamma_\mu, \tag{S3}$$

since this would entail attempting to estimate more coefficients than there are data points ($m = 213$ descriptors and the data set contains $n = 146$ polymers). In addition, many descriptors in $\mathbf{D}$ are strongly correlated (Fig. S5), resulting in multicolinearity between predictors in the design matrix $\bar{\mathbf{X}}\mathbf{D}$. As demonstrated by the scree plots in Fig. S6, the fact

that less than 15 principal components have non-negligible variance for a data set containing a total of 213 descriptors, explains the significant multicollinearity. We therefore employ alternative regression methods, which each introduce a small amount of bias in order to improve the prediction accuracy and address this multicolinearity problem. The used methods are: Ridge Regression, Lasso Regression, Principal Component Regression (PCR) and Partial Least Squares (PLS) Regression [5].

A. Standardising variables

The regression methods used in this study (PCA, PLS, Ridge and Lasso) are not scale-invariant and solutions can thus differ depending on the units of the inputs. To avoid this, we standardise the inputs to have zero mean and a sample variance of one. The standardised input matrix $\mathbf{R}^*$ is calculated as

$$R^*_{a\mu} = \frac{R_{a\mu} - \overline{R_\mu}}{s_{R_\mu}}, \qquad \text{where} \qquad R_{a\mu} = \sum_{i=1}^{p} \bar{X}_{ai} D_{i\mu} \equiv (\bar{\mathbf{X}}\mathbf{D})_{a\mu}, \tag{S4}$$

with sample mean $\overline{R_\mu}$ and variance $s^2_{R_\mu}$ for descriptor μ given by

$$\overline{R_\mu} = \frac{1}{n}\sum_{a=1}^{n} R_{a\mu}, \qquad s^2_{R_\mu} = \frac{1}{n-1}\sum_{a=1}^{n}(R_{a\mu} - \overline{R_\mu})^2. \tag{S5}$$

Similarly, $T_{\rm g}{}^a$ is standardised,

$$T_{\rm g}^{*a} = \frac{T_{\rm g}{}^a - \overline{T_{\rm g}}}{s_{T_{\rm g}}}, \tag{S6}$$

with sample mean and variance given by

$$\overline{T_{\rm g}} = \frac{1}{n}\sum_{a=1}^{n} T_{\rm g}{}^a, \quad s^2_{T_{\rm g}} = \frac{1}{n-1}\sum_{a=1}^{n}(T_{\rm g}{}^a - \overline{T_{\rm g}})^2. \tag{S7}$$

The standardised fitting function is redefined as

$$f((\mathbf{R}^*)_a) = \sum_{\mu=1}^{m} R^*_{a\mu}\gamma^*_\mu, \tag{S8}$$

where γ^*_μ are the standardised regression coefficients for $\mu = 1, \ldots, m$. Once estimated (see below), the regression coefficients $\hat{\gamma}^*_\mu$ can be converted back to the original unstandardised regression coefficient estimations $\hat{\gamma}_\mu$ and $\hat{\gamma}_0$ by

$$\hat{\gamma}_\mu = \frac{s_{T_{\rm g}}}{s_{R_\mu}}\hat{\gamma}^*_\mu, \quad \hat{\gamma}_0 = \overline{T_{\rm g}} - \sum_{\mu=1}^{m}\hat{\gamma}_\mu \overline{R_\mu}. \tag{S9}$$

B. Shrinkage methods

In Ordinary Least Squares the standardised glass transition temperature $T_{\rm g}^{*a}$ is taken to be linearly related to the standardised matrix $\mathbf{R}^*$, and a least squares minimisation is performed to estimate the standardised regression coefficients $\hat{\boldsymbol{\gamma}}^*$ according to

$$\hat{\gamma}^*_{OLS} = \arg\min_{\boldsymbol{\delta}} \left\{ \sum_{a=1}^{n}[T_{\rm g}^{*a} - \sum_{\mu=1}^{m} R^*_{a\mu}\hat{\gamma}^*_\mu]^2 \right\}. \tag{S10}$$

We note that for the standalone QSPR method, the matrix $\mathbf{R}^*$ instead represents the set of descriptors derived directly from the 3D molecule corresponding to the monomer unit. The descriptors are first normalised by the monomeric mass

and then standardised to give the matrix $\mathbf{R}^{*}$. This standardised QSPR matrix equivalently applies in the regression model above and those that follow below.

For Ridge and Lasso regression methods an additional term (a penalty) with a value related to the size of $\boldsymbol{\gamma}^{*}$ is incorporated in the function to be minimised. The presence of this term penalises the size of the estimated coefficients, resulting in many fewer 'effective' regression coefficients; this process is known as shrinkage. In Ridge regression the added penalty is proportional to the L2-norm of the coefficients, whereas for Lasso regression, the penalty is proportional to the L1-norm of the coefficients, as shown below:

$$\hat{\gamma}^{*}_{Ridge} = \underset{\boldsymbol{\gamma}^{*}}{\arg\min} \left\{ \sum_{a=1}^{n} [T_{\mathrm{g}}^{*a} - \sum_{\mu=1}^{m} R^{*}_{a\mu}\gamma^{*}_{\mu}]^2 + \alpha \sum_{\mu=1}^{m} (\gamma^{*}_{\mu})^2 \right\}, \tag{S11a}$$

$$\hat{\gamma}^{*}_{Lasso} = \underset{\boldsymbol{\gamma}^{*}}{\arg\min} \left\{ \sum_{a=1}^{n} [T_{\mathrm{g}}^{*a} - \sum_{\mu=1}^{m} R^{*}_{a\mu}\gamma^{*}_{\mu}]^2 + \alpha \sum_{\mu=1}^{m} |\gamma^{*}_{\mu}| \right\}, \tag{S11b}$$

where the hyperparameter α controls the degree of applied coefficient shrinkage. If α is sufficiently large, then for Lasso regression (L1) some coefficients will shrink to exactly zero, whereas for Ridge regression, some coefficients will shrink to values close to zero but never actually reach zero; this is due to the nature of each penalty term (for further information, see [5]). As α approaches zero, both Ridge and Lasso regression will converge to the OLS solution. The hyperparameter α is optimised during internal validation, which is discussed further in Section S-III.

Kernel Ridge regression (KRR) is used as one of the benchmarking QSPR models. Like Ridge, this applies an L2 regularisation on the fit; however, it also makes use of a *kernel* which provides a means of introducing non-linearity into the model. A brief introduction follows to clarify the notation for the hyperparameters, and for further details see [5, 6]. A kernel $K(\boldsymbol{r}^{*}_{a}, \boldsymbol{r}^{*}_{b})$ is a function that captures the similarity between two objects. In the case of the QSPR model, the two objects are m-dimensional descriptor vectors that encode their corresponding polymers, denoted $\boldsymbol{r}^{*}_{a}$ and $\boldsymbol{r}^{*}_{b}$; each vector represents rows a and b in the standardised polymer-descriptor matrix $\mathbf{R}^{*}$, respectively. Typically $K(\boldsymbol{r}^{*}_{a}, \boldsymbol{r}^{*}_{b})$ is a symmetric real-valued function (i.e., $K(\boldsymbol{r}^{*}_{a}, \boldsymbol{r}^{*}_{b}) = K(\boldsymbol{r}^{*}_{b}, \boldsymbol{r}^{*}_{a})$) which usually satisfies $K(\boldsymbol{r}^{*}_{a}, \boldsymbol{r}^{*}_{b}) \geq 0$, supporting its interpretation as a similarity measure [6]. Among the many kernels available, the one chosen in this study is the Radial Basis Function (RBF) kernel, defined

$$(\mathbf{K})_{ab} = K(\boldsymbol{r}^{*}_{a}, \boldsymbol{r}^{*}_{b}) = \exp\bigl(-\gamma \|\boldsymbol{r}^{*}_{a} - \boldsymbol{r}^{*}_{b}\|^2\bigr), \tag{S12}$$

where $\mathbf{K}$ is an $n \times n$ symmetric positive semi-definite matrix. KRR solves

$$\hat{\boldsymbol{c}}^{*} = \underset{\boldsymbol{c}^{*}}{\arg\min} \left\{ \|\boldsymbol{T}^{*}_{\mathbf{g}} - \mathbf{K}\,\boldsymbol{c}^{*}\|^2 \;+\; \alpha\,\boldsymbol{c}^{*\top}\mathbf{K}\,\boldsymbol{c}^{*} \right\}, \tag{S13}$$

where the coefficients $\boldsymbol{c}^{*} \in \mathbb{R}^{n}$. Hyperparameters α and γ control the degree of shrinkage and the smoothness of the unknown, and thus estimated, function $f(\mathbf{r})$ that returns a T_{g} prediction for a given descriptor vector $\boldsymbol{r}$. The estimated function is given by $\hat{f}(\mathbf{r}) = \sum_{a=1}^{n} \hat{c}^{*}_{a}\, K\bigl(\boldsymbol{r}, \boldsymbol{r}^{*}_{a}\bigr)$.

C. PCR and PLS dimension reduction methods

Both Principal Component Regression (PCR) and Partial Least Squares (PLS) regression consist of a transformation of the standardised input data $\mathbf{R}^{*}$, and the application of a regression procedure. PCR executes these steps separately, while PLS performs them simultaneously. Both regression methods use the *singular value decomposition* (SVD) factorisation on the non-square $n \times m$ matrix $\mathbf{R}^{*}$. If $\mathbf{R}^{*}$ has rank r then it has r non-zero singular values, which equivalently correspond to the r non-zero (square roots of) eigenvalues of the matrix $\mathbf{R}^{*\top}\mathbf{R}^{*}$. The SVD of $\mathbf{R}^{*}$ is given by $\mathbf{R}^{*} = \mathbf{U}\,\mathbf{S}\,\mathbf{V}^{\top}$ where $\mathbf{U}_{n\times r}$ and $\mathbf{V}_{m\times r}$ are respectively the sets of left and right singular vectors of $\mathbf{R}^{*}$, and $\mathbf{S}_{r\times r}$ is the diagonal matrix of singular values, ordered from largest to smallest.

PCR transforms and compresses $\mathbf{R}^{*}$ onto its first k principal components ($k \leq r$), using only the first $1, \ldots, k$ columns of $\mathbf{V}$, denoted $\mathbf{V}_k$ ($m \times k$). The transformation $\mathbf{R}^{*}\,\mathbf{V}_k$ drops the $(k+1), \ldots, m$ columns of $\mathbf{R}^{*}\,\mathbf{V}$ that explain the smallest sample variance (corresponding to the smallest eigenvalues of $\mathbf{R}^{*\top}\mathbf{R}^{*}$). Then, a least squares minimisation is performed on the transformed matrix $\mathbf{R}^{*}\,\mathbf{V}_k$ with k regression coefficients $\boldsymbol{\xi} \in \mathbb{R}^{k}$, yielding the estimator

$$\hat{\boldsymbol{\xi}} = \underset{\boldsymbol{\xi}}{\arg\min} \|\boldsymbol{T}^{*}_{\mathbf{g}} - \mathbf{R}^{*}\,\mathbf{V}_k\,\boldsymbol{\xi}\|^2. \tag{S14}$$

The first k components are referred to as $n_{\text{components}}$ in Table S2. The solution to Eq. (S14) gives

$$\hat{\boldsymbol{\xi}} = \mathbf{S}_k^{-1}\,\mathbf{U}_k^\top\,\boldsymbol{T}_{\mathbf{g}}^*, \tag{S15}$$

since $\mathbf{R}^*\,\mathbf{V}_k = \mathbf{U}_k\,\mathbf{S}_k$, where $\mathbf{S}_k$ is the diagonal matrix of the largest k singular values $(k \times k)$ and $\mathbf{U}_k$ is the set of left singular vectors $(n \times k)$. Importantly, the reduced standardised coefficient estimates $\hat{\boldsymbol{\gamma}}^*_{PCR}$ are determined by the transformation $\hat{\boldsymbol{\gamma}}^*_{PCR} = \mathbf{V}_k\,\hat{\boldsymbol{\xi}}$ [7]. PCR will perform poorly if $\boldsymbol{T}_{\mathbf{g}}^*$ is strongly correlated in directions with small variance, *i.e.* principal components that are strongly related to T_{g}, but have small singular values.

PLS does not suffer this issue since it includes $\boldsymbol{T}_{\mathbf{g}}^*$ in the data compression process. Like PCR, the PLS algorithm compresses the data to the first k components ($n_{\text{components}}$ in Table S2), but now using the first k left and right singular vectors from the SVD of the inner product of $\mathbf{R}^{*\top}\,\boldsymbol{T}_{\mathbf{g}}^*$. We use the formulation of PLS from the Python module Scikit-learn [8], which is implemented from Wegelin [9].

D. Genetic algorithm

A genetic algorithm employs concepts inspired by biology to solve optimisation problems, utilising processes such as natural selection, random mutation, and genetic recombination [10]. We follow the procedure outlined in Fig. S7 [11]. In the present study, a chromosome is represented as a binary bit string (Fig. S8) of $m = 213$ genes, where the μth gene corresponds to the μth descriptor. The value 1 signifies that a gene (descriptor) is 'turned on', while the value 0 means that a gene (descriptor) is not turned on.

The algorithm begins with a set of initial conditions, as shown in Table S1, the first being *population size*. A population consists of a number of chromosomes, where each chromosome is represented by a sequence of randomly distributed 1's and 0's (genes). The population size is set to be equal to the size of the gene pool, *i.e.* the number of genes in a single chromosome. This enables enough variation of genes to represent the full set of descriptors when the population is initialised. Despite the random distribution of 1's and 0's throughout a chromosome, all chromosomes are constrained to have a fixed number of m_{GA} active genes (value 1). For example, QSPR-GAP GA2 always contains 2 genes with value 1, and the remaining 211 genes have value 0.

The initial population is evaluated for each individual chromosome's *fitness*. The fitness is evaluated using a fitness function which is determined during internal validation (see Fig. S12). The genes are indexed according to the molecular descriptors μ. When the μth gene $= 1$, then the μth descriptor is incorporated into a linear regression model. When more than one gene is turned on (equal to 1), then the corresponding descriptors are used in a multiple linear regression; specifically, the robust regression model `sklearn.linear_model.HuberRegressor` was used (with Scikit-learn's default hyperparameters). To determine the fitness, consider a single training-test split for a single chromosome within the internal validation. Through some random process a set of m_{GA} descriptors have been 'activated' within the chromosome. These descriptors are then included in the regression model, where the training set is used to fit the model and predictions are made on the test set (still within the internal validation). Once the prediction has been made the root-mean-squared-error (RMSE) is calculated between the predictions and test data (see Eq. S16). The fitness of the ith chromosome was defined as $f_i = 1/(\text{RMSE})_i$ and every chromosome is assigned a fitness values accordingly.

Based on the fitness of the chromosomes, 50% of the population is then *selected* to have their genes passed on to the next generation. The mechanism used was **roulette wheel selection** [10, 11] (RWS) which introduces a stochastic element to the selection process. Given a population of N chromosomes, the probability of selecting the ith chromosome is distributed according to $p_i = f_i / \sum_{i=1}^{N} f_i$. The next generation is created by sampling $N/2$ chromosomes according to this distribution with replacement; *i.e.* the same chromosome can be sampled more than once for the next generation.

The genetic recombination step, known as *crossover*, consists of two parent chromosomes mixing their genes into two child chromosomes, but with each parent chromosome undergoing crossover with two different parent chromosomes to yield two children for every parent, on average. In the example shown in Fig. S9, Parents 1 and 2 (P1 and P2) crossover to make children 1 and 2 (C1 and C2). However P1 also crosses over with P8 to make C15 and C16, while P2 crosses over with P3 to make C3 and C4. In this manner the population doubles, which exactly counteracts the halving of the population that occurred during the selection phase. To ensure that the total number of genes equal to 1 (turned on) in a given chromosome (m_{GA}) remains constant, the crossover mechanism adheres to the method illustrated in Fig. S9. For illustrative purposes, we only show ten genes per chromosome in Fig. S9 (when actually there are 213 genes). The crossover process is governed by the following rules: when the μth gene is the same for both P1 and P2 (as indicated by the red boxes around the genes) then this 'strong' μth gene passes on to C1 and C2 with probability 1. For the remaining genes which do not match, these are (randomly) shuffled in any order as shown in

the figure. This process guarantees the m_{GA} 'turned on' genes remains the same in both C1 and C2 as was previously in P1 and P2.

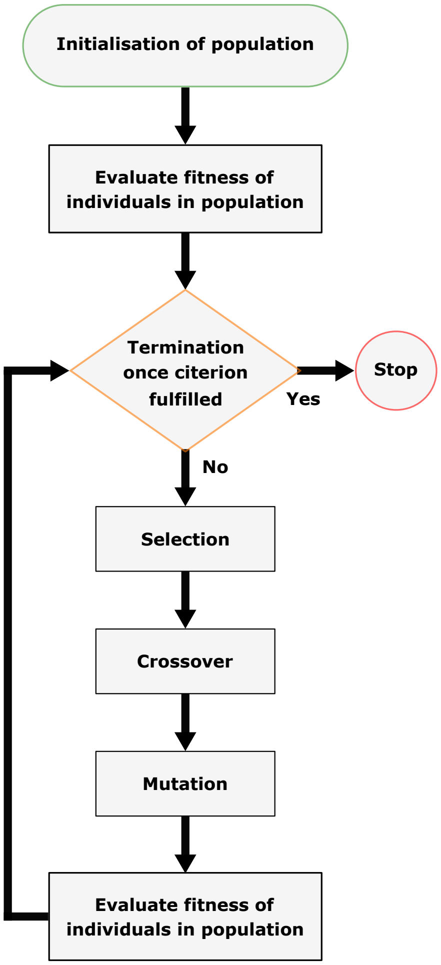

Figure S7: **Genetic algorithm flow diagram,** starting with an initialisation of a population and ending with a termination criterion. Each step is discussed in the current section and the parameters accompanying each step can be found in Table S1.

GA parameter	Value
Population size	213 chromosomes
Gene pool	213 genes (molecular descriptors)
Number of generations	50 generations
`mutation_rate_per_pop`	probability $= 0.1$
`mutation_rate_per_chrom`	proportion $= 0.9/m_{\mathrm{GA}}$

Table S1: **Parameter inputs for the genetic algorithm.** Population size is measured in the number of chromosomes, which is taken to match the size of the gene pool, and the algorithm terminates after the number of generations. The mutation rate per population, given by `mutation_rate_per_pop`, defines the probability a chromosome will be mutated in the population (in a given generation). The mutation rate per chromosome is defined by a randomly selected subset of genes that are randomly shuffled. The value of `mutation_rate_per_chromosome` defines the ratio of this subset size to the size of the chromosome.

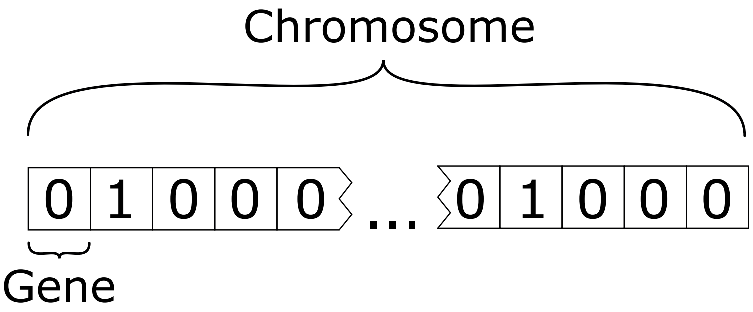

Figure S8: **Binary representation of a chromosome.** Chromosomes used in the present study contain 213 genes, where the μth gene takes values 0 (turned off) or 1 (turned on) and corresponds to the μth descriptor/feature $(\mathbf{D})_\mu$.

Once the next generation has been created, the resulting child chromosomes are *mutated* to ensure diversity through the population and prevent premature convergence to local optima. The probability of mutation is controlled by two parameters: `mutation_rate_per_pop` and `mutation_rate_per_chrom` which both take values between 0 and 1. The former controls the probability that a chromosome in the population will be mutated during a given round of mutation. If `mutation_rate_per_pop` $= 0.5$ then there is a 50% chance a given chromosome will be mutated. The latter controls the proportion of genes in the chromosome that will be randomly shuffled; *i.e.* if `mutation_rate_per_chrom` $= 0.1$ then 21 randomly chosen genes will be randomly shuffled, out of the total 213 genes in a chromosome. Since the chromosomes are dominated by zeros ($m_{\mathrm{GA}} \leq 10$), the selection defined by `mutation_rate_per_chrom` will often just shuffle zeros and not change the chromosome. Hence, the effective mutation rate is roughly proportional to the number of genes (m_{GA}) that have been turned on. To counter this, we have assumed that `mutation_rate_per_chrom` is inversely proportional to m_{GA}, to enhance the possibility of mutations when just a few genes are turned on (Table S1); this is an attempt to maintain a level of independence between the mutation rate per chromosome and the total number of active genes m_{GA}.

Finally, the fitness of the new generation is evaluated and the process repeats until *termination*, which we have specified as after 50 generations. At this point, the best solution (*i.e.* chromosome with best fitness) from all 50 generations was used for the model. The set of optimised descriptors corresponding to the best (fittest) chromosome

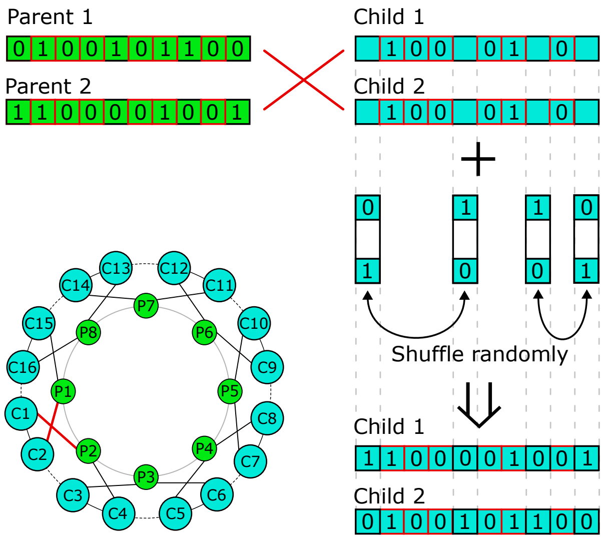

Figure S9: **The crossover operation**. In this process each parent chromosome combines with *two* other parents to produce a total of two child chromosomes per parent. The top and the right show P1 and P2 sharing genes to children C1 and C2. The crossover wheel (bottom left) indicates how the crossover operation is applied to the population as a whole, so that P1 crosses over with P8 (creating C15 and C16) as well as P2, while P2 crosses over with P3 (creating C3 and C4) as well as with P1. This doubles the population.

were used in the *external validation*, in which those optimised descriptors were fit to the training set to finally make predictions about the test data (see Fig. S12).

S-III. MODEL EVALUATION

For all models, we perform an **external validation** and an **internal validation**. The goal of the external validation is to predict polymer T_g's that were not used to determine/train the model parameters ('out-of-sample data'), while the goal of the internal validation was to tune hyperparameters for a given model, or to select the optimal set of descriptors. For clarity, we refer to the PCR, Ridge, Lasso, PLS and KRR models as the **'Statistical models'**; and models that use descriptor selection based on a genetic algorithm as **'Genetic Algorithm Models'.** The internal validation was conducted differently for these two sets of approaches (see Fig. S10 and Fig. S12), but the external validation was the same for both. For the GAP models, no internal validation was required.

The external validation was performed using a repeated five-fold cross validation (5-fold CV), where the full data set was shuffled randomly and subsequently partitioned into five exclusive subsets. A test set was iteratively selected from the 5 subsets and, in each iteration, the remaining four subsets were concatenated into a training set. The partitioning into five subsets was repeated 10 times. This procedure resulted in a total of 50 different train-test splits with 50 different combinations of polymers in the training and test sets. For every train-test split, the test set was left out and the internal validation was conducted on the remaining training data set, where the models were tuned and optimised.

The performance metric used for validation (both internal and external) was the root-mean-squared error of the test data, given by

$$\mathrm{RMSE} = \sqrt{\frac{\sum_{b=1}^{B}({T_g}^b - \hat{T_g}^b)^2}{B}}, \tag{S16}$$

where $b = 1, \ldots, B$ indexes over the polymers in the test set. ${T_g}^b$ is the experimental T_g for the bth polymer and $\hat{T_g}^b$ is an out-of-sample T_g prediction made on the bth polymer.

A. Statistical models

For the internal validation of the statistical models, hyperparameter tuning was performed using a one dimensional exhaustive grid search with Scikit-learn's function `sklearn.model_selection.GridSearchCV`. The grids of values for each hyperparameter are listed in Table S2. During the internal validation, the training data was split into five folds (Fig. S10). All discrete values defined in Table S2 were applied (as appropriate for each model) using 4 folds to fit the model (the internal training set) and evaluated on the remaining fold (internal test set). This process was repeated for each of the five folds, and the hyperparameter which yielded the best performance (lowest RMSE) of the five folds was selected for the external validation. The optimised hyperparameters for all 50 training-test splits are presented in Fig. S11; these represent the best hyperparameters selected from the 50 internal validations, which were subsequently used for the external validation.

Estimator	Hyperparameters	Range	Python Command
PCR	$n_{\text{components}}$	Integers $\mathbb{Z} = 1, \ldots, 20$	`list(range(1, 21))`
Ridge	α	$\log_{10} \alpha \in [-3, 3)$ in 21 steps	`list(numpy.logspace(-3, 3, 21))`
Lasso	α	$\log_{10} \alpha \in [-3, 3)$ in 21 steps	`list(numpy.logspace(-3, 3, 21))`
PLS	$n_{\text{components}}$	Integers $\mathbb{Z} = 1, \ldots, 20$	`list(range(1, 21))`
KRR	α	$\log_{10} \alpha \in [-8, 4)$ in 7 steps	`list(numpy.logspace(-8, 4, 7))`
	γ	$\log_{10} \gamma \in [-8, 4)$ in 7 steps	`list(numpy.logspace(-8, 4, 7))`

Table S2: **Candidate hyperparameter values for grid search implementation.**

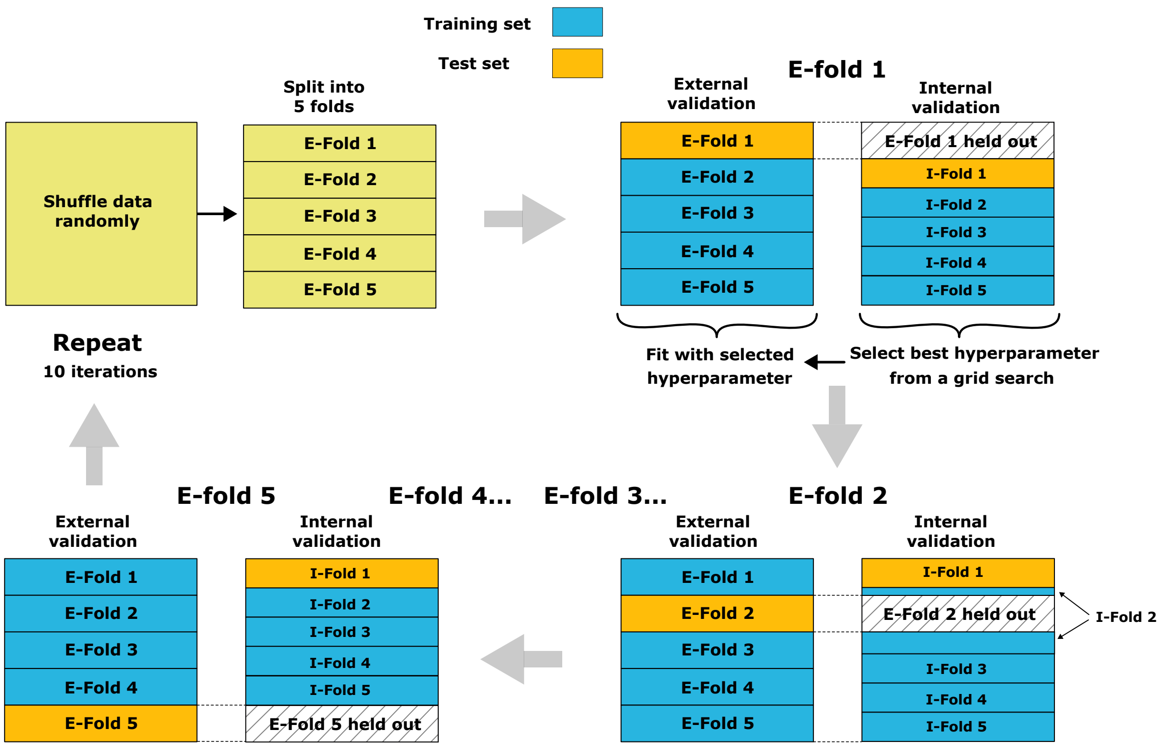

Figure S10: **Model validation for the 'statistical models'.** The key take-away of the figure is the partitions of the data during external and internal validation. When a test set is selected (iteratively) during external validation, this test set is **held out** from the internal validation as indicated by the diagram. The optimal hyperparameters are selected through internal validation, after which they are used for the external validation. In this phase (external), the model is fit with the training data (and optimised hyperparameters), and used to predict the out-of-sample (test) data; the performance accuracy measure used is the RMSE (Eq. S16).

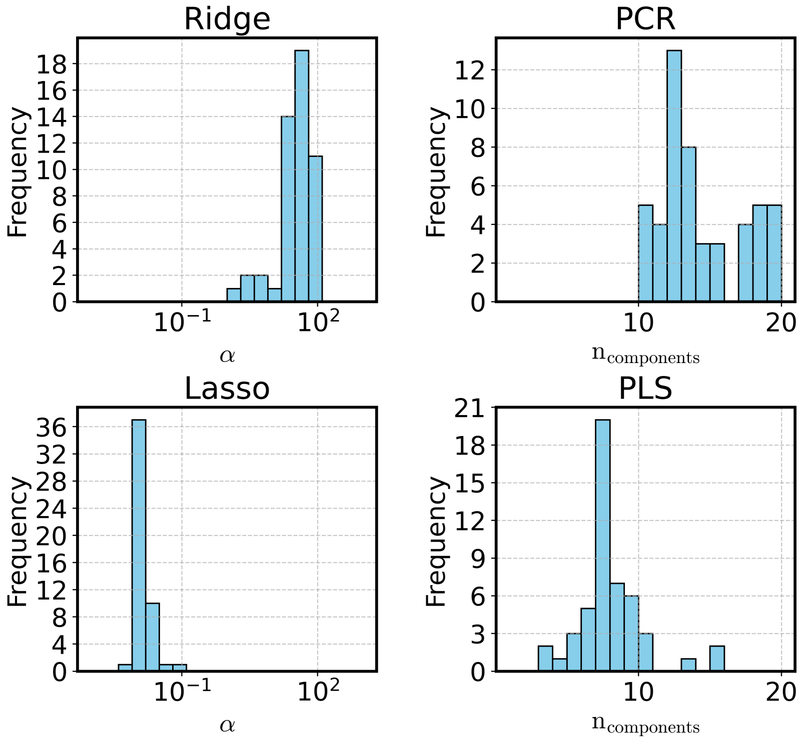

(a) **Hyperparameter distributions for the QSPR-GAP models.**

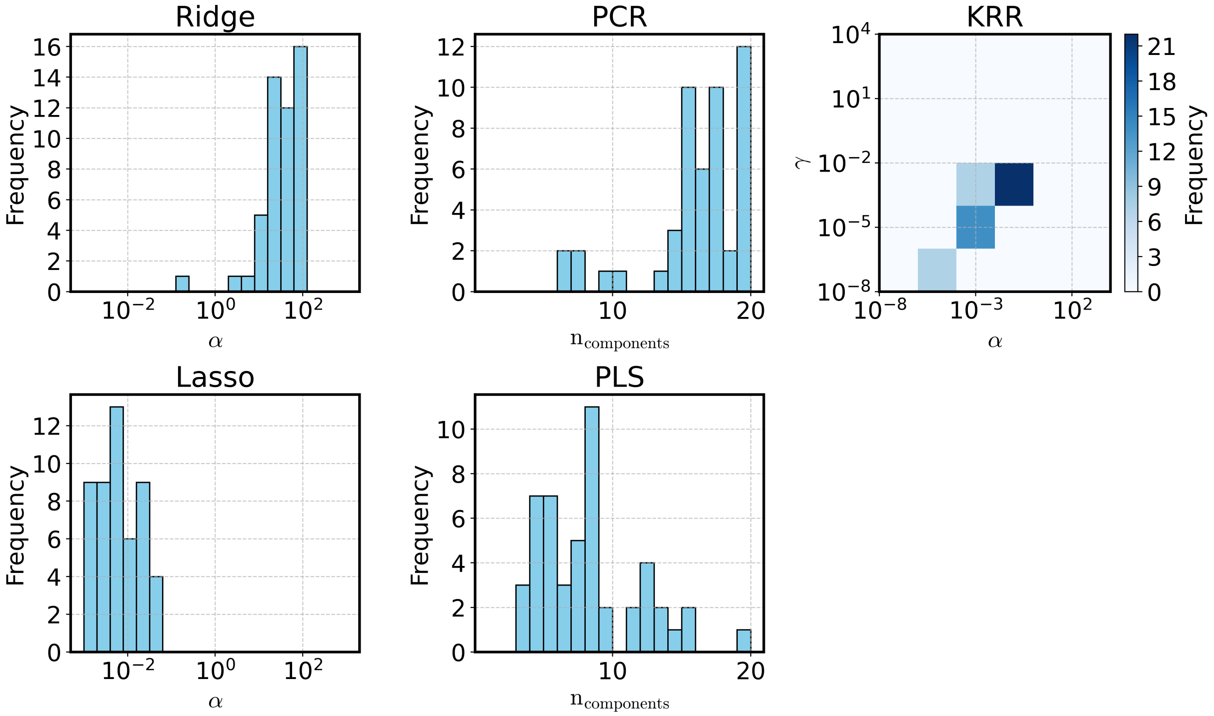

(b) **Hyperparameter distributions for the QSPR models.**

Figure S11: **Distributions of optimised hyperparameters,** showing the number of occasions a hyperparameter is picked from the range in Table S2. The hyperparameters are selected based on the optimisation process during internal validation. For a given training-test split (in the external validation), the optimal hyperparameter is selected by applying the internal validation on the external training set. Overall there are 50 splits, and thus 50 different optimal hyperparameters (for the different data partitions). We show the distribution of these 50 hyperparameters here.

B. Genetic algorithm models

For the GA models, the internal validation included 50% training and 50% test data selected at random (Fig. S12). The GA was applied during the internal validation; the fitness of the chromosomes was evaluated by fitting to the internal training set and predicting the internal test set, using the inverse RMSE as the fitness metric. Once the best chromosome was determined by maximising the fitness, the set of descriptors corresponding to the best chromosome were used to fit the model to the training data in the external validation, and to predict the test data. This process was repeated a total of 50 times as depicted in Fig. S12. The evolution of fitness through each generation was recorded for all 50 of the internal validations conducted. We show an example of five from the 50 internal validations in Fig. S13 for the ten different QSPR-GAP GAm_{GA} models ($m_{\mathrm{GA}} = 1, \ldots, 10$).

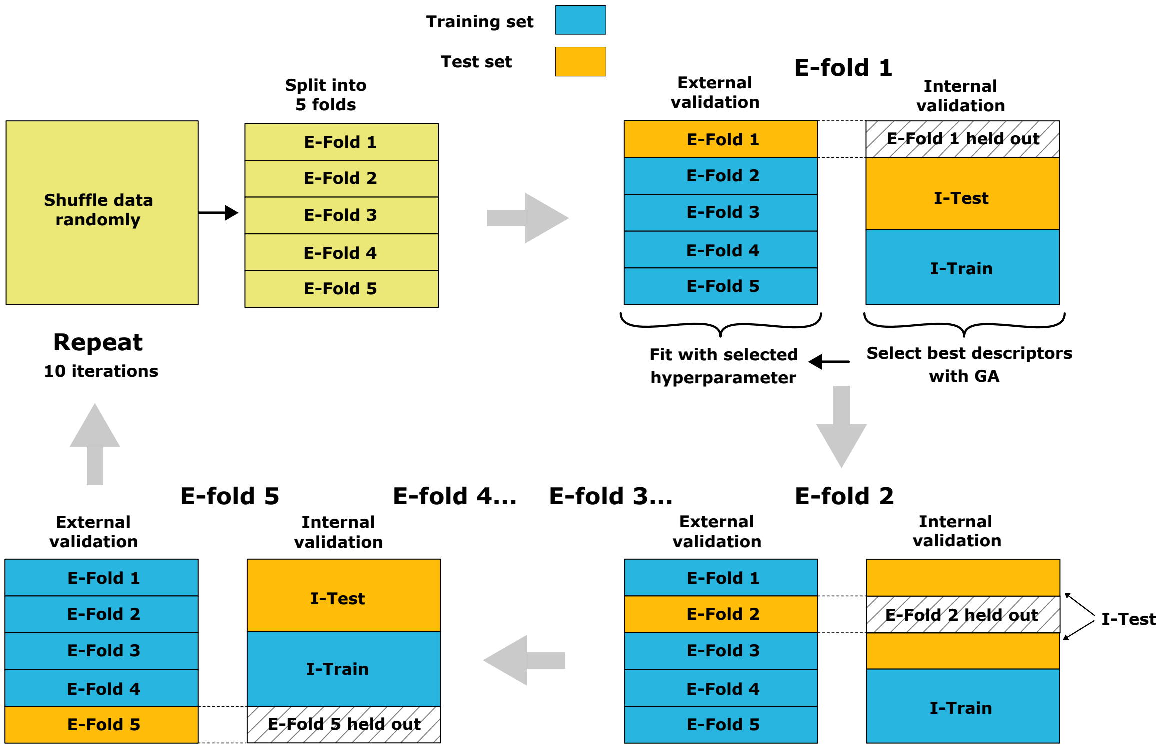

Figure S12: **Model validation for the 'genetic algorithm models'.** The division of training and test sets in both external and internal validations is shown. For a given external training set, a two-way split of internal training and test data was used to calculate the fitness (inverse RMSE). In total there are 50 different two-way splits, each representing a unique internal validation. Within each internal validation, the genetic algorithm evolved over 50 generations of chromosomes to optimise the fitness (see Fig. S13). The internal validation thus outputs the fittest chromosome (lowest RMSE), corresponding to the optimal m_{GA} descriptors. These descriptors were subsequently used for the external validation, predicting the test data from the training data with OLS; the predictive accuracy was measured with the RMSE (Eq. S16).

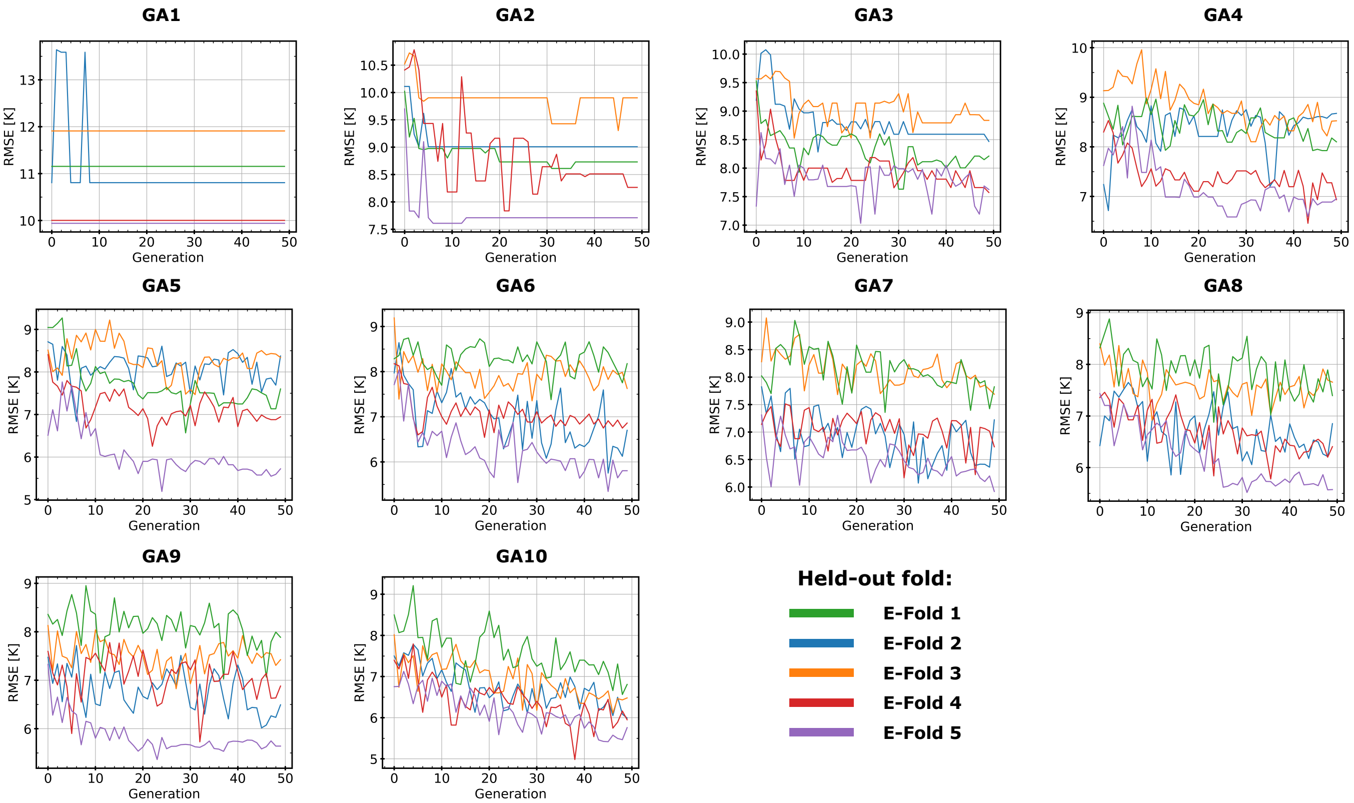

Figure S13: **Evolution of chromosomes for all genetic algorithm models.** The RMSE of the best chromosome per population is shown through each generation. The RMSE is calculated in the internal validation (Fig. S12) and the chromosome with the lowest RMSE (from all generations) is then applied in the external validation to predict the held-out fold (shown by the plot legend). All ten GAm_{GA} models are presented, where m_{GA} is the number of features/descriptors used in each model (the number of genes 'turned on' to 1 in a chromosome).

C. Summary of results

To accompany the results in the main text, we include additional information from the model validation stage. Fig. S14 shows the number of out-of-sample fragment occurrences from the 50 different combinations of polymers in the training and test set during the repeated 5-fold CV. The various definitions of fragments result in different numbers of out-of-sample fragment occurrences: *L-Ar* has fewer occurrences since there are fewer unique fragments, while *Ar-L-Ar* has the most occurrences.

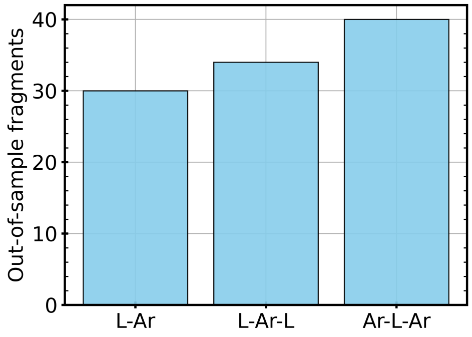

Figure S14: **Number of out-of-sample fragment occurrences during model validation.** An out-of-sample fragment occurrence is when the data set of polymers is split into training and test sets randomly, and there happens to be at least one polymer in the test set which is made up of at least one fragment that does not exist in the training set.

The comparative plots of QSPR-GAP vs GAP in the model performance evaluation (Fig. 2 of the main text) demonstrate how the GAP approach suffers for predictions of polymers containing out-of-sample fragment occurrences. Fig. S15a shows how the models compare when the training-test splits containing out-of-sample fragment occurrences have been removed. This is the same data presented in Fig. 2d of the main text, except that the RMSEs calculated from training-test splits containing any out-of-sample occurrences have been removed from the plot.

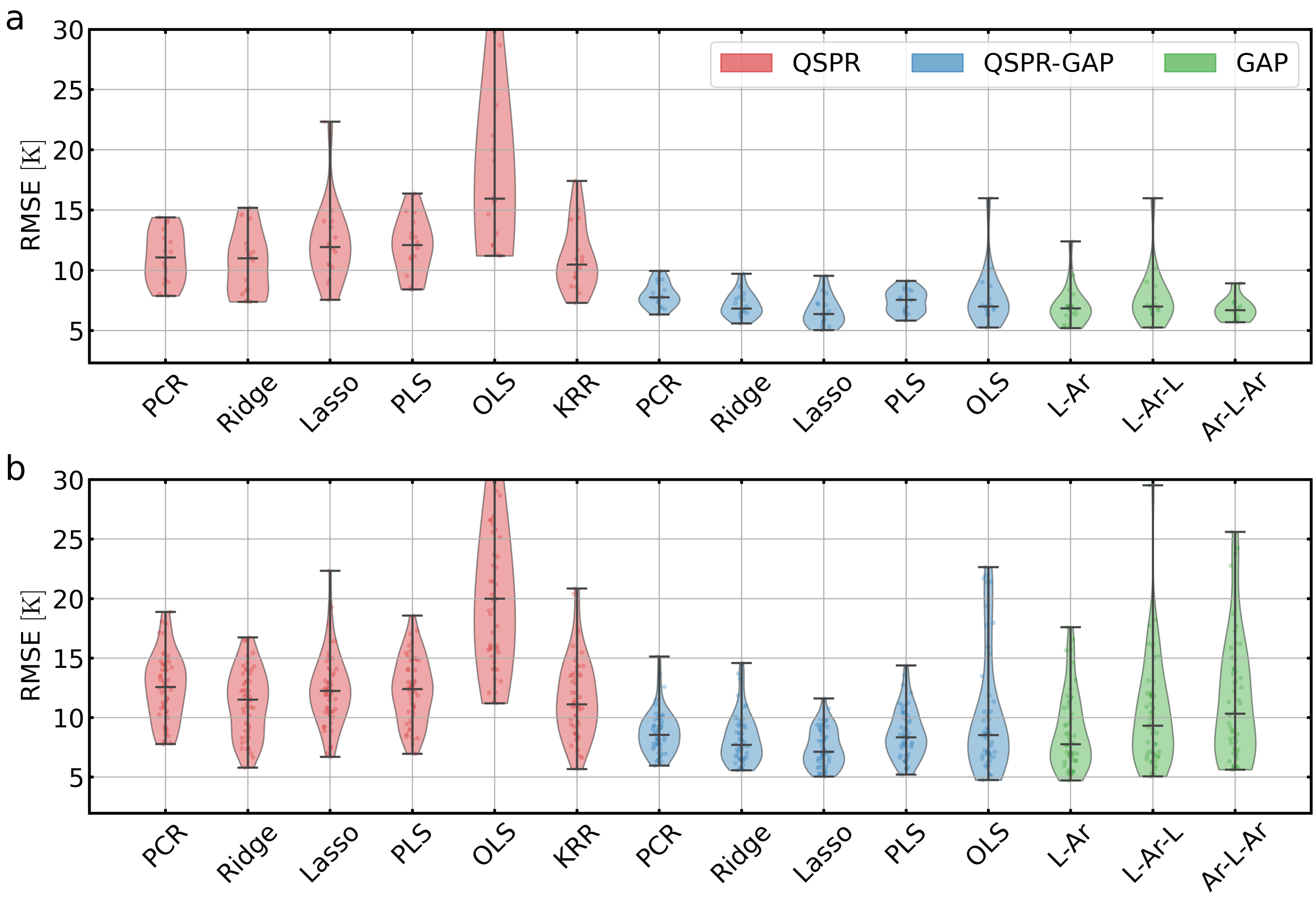

Figure S15: **Results of the QSPR vs. the QSPR-GAP model vs. the GAP model with all out-of-sample fragment occurrences removed.** A repeated 5-fold cross validation scored by root-mean-squared error (RMSE). **(a)** corresponds to Fig. 2d in the main text with out-of-sample fragment occurrences removed. **(b)** shows Fig. 2d in the main text again (without the removal of out-of-sample fragment occurrences). Note that the out-of-sample fragment occurrences removed in all the QSPR and QSPR-GAP models correspond to the *L-Ar-L* type. Interestingly, based on the prediction results, the OLS QSPR-GAP model produces identical results to the GAP *L-Ar-L*. This is likely because, even though the QSPR-GAP descriptor matrix ($D_{i\mu}$) has 213 columns, its rank ($p = 30$) is equal to that of the GAP composition matrix ($\bar{X}_{ai}$) when there are no out-of-sample fragment occurrences.

S-IV. FRAGMENT CONTRIBUTIONS $\hat{\beta}_i$

The T_g contributions $\hat{\beta}_i$ from each fragment i is shown in Fig S17 for the following models: GAP, QSPR-GAP Lasso and QSPR-GAP GA2. The values of $\hat{\beta}_i$ are tabulated in Table S5 for all models applied to the *L-Ar-L* fragments; the regressions were performed on the full data set of 146 polymers. The fragments are ordered from smallest to largest $\hat{\beta}_i$ values according to the QSPR-GAP GA2 model. Hyperparameters for the models PCR, Ridge, Lasso and PLS were determined from a 5-fold CV and the hyperparameter which yielded the best performance (lowest RMSE) of the five folds was selected. The optimal features (or descriptors) selected by the genetic algorithm was determined from the analysis in the main text. The hyperparameters and optimal features used to calculate the data in Fig. S17 are found in Table S3.

**Estimator**	**Hyperparameters**
PCR	$n_\mathrm{components} = 13$
Ridge	$\alpha = 63.10$
Lasso	$\alpha = 0.007943$
PLS	$n_\mathrm{components} = 7$
GA2	descriptors = `Mor05m` and `Mor26m`
GAP	N/A

Table S3: **Hyperparameters for the models in Table S5.**

Diagnostic plots are shown in Fig. S16 to assess the assumption of normality for the residuals, which unless the sample size is sufficiently large, is required to report the inference in Table S5, and in Table I of the main text. The QSPR-GAP GA2 model with descriptors `Mor05m` and `Mor26m` shows a single extreme outlier with repeating unit structure: PENEPKDK [12] (see Fig. S2 for the corresponding chemical structure). The clear outlier in the plots has a Cook's distance of 0.12, and for further testing of it's influence, coefficient estimates and confidence intervals were determined from the data sample with the outlier removed. Table S4 shows the coefficient estimates and confidence intervals when the outlier is included in the fit (*i.e.* fit to the full data sample of 146 polymers) and when the outlier is removed from the sample and fit to the remaining 145 polymers. Since the confidence intervals and parameter estimates are weakly influenced by this outlier, and given the large sample size (large number of observations per variable), the normality assumption of residuals is not required in this case. However, for the GAP model, the number of non-zero observations per predictor is not constant (see Fig. S4); there are as few as one non-zero observation for certain predictors. We report the confidence intervals for the GAP model in Table S5 however they should be interpreted with caution, given the slight deviation from linearity in the Q-Q plots shown in Fig. S16 at the extremes of the residuals.

μ	Descriptor	$\hat{\gamma}_\mu$ [K] incl. outlier	CI (95%) L/U [K] incl. outlier	$\hat{\gamma}_\mu$ [K] w/o outlier	CI (95%) L/U [K] w/o outlier
0	–	298	286/310	296	284/307
1	`Mor05m`	-58	-67/-50	-60	-68/-52
2	`Mor26m`	-198	-239/-157	-193	-231/-155

Table S4: **Influence of the outlier on parameter estimations**. The table shows the QSPR-GAP GA2 model with descriptors `Mor01m` and `Mor26m` fit to the full data sample of 146 polymers; coefficient estimates and confidence intervals (CIs) are shown under 'incl. outlier'. The table also presents this model fit to the data sample of 145 polymers with the outlier removed; coefficient estimates and CIs are shown under 'w/o outlier'.

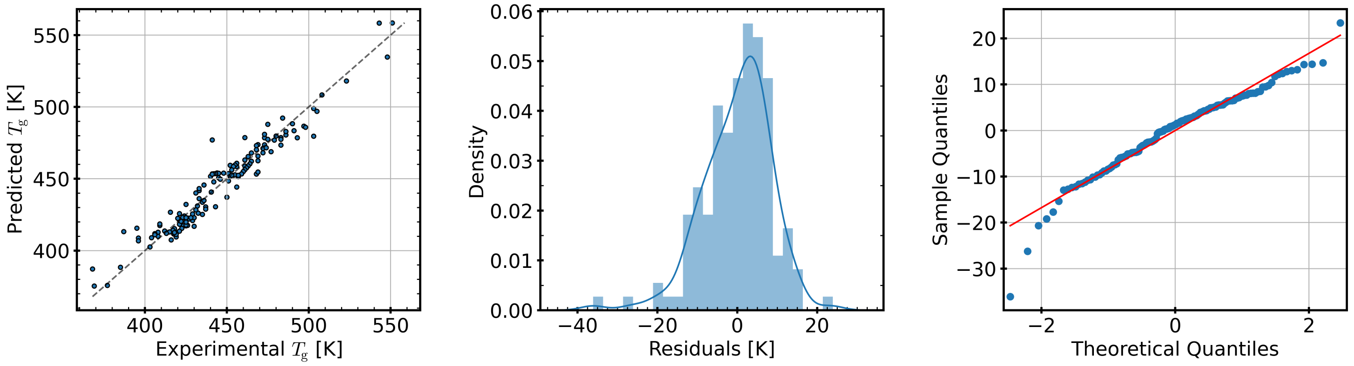

(a) **QSPR-GAP GA2 model with Mor05m and Mor26m.**

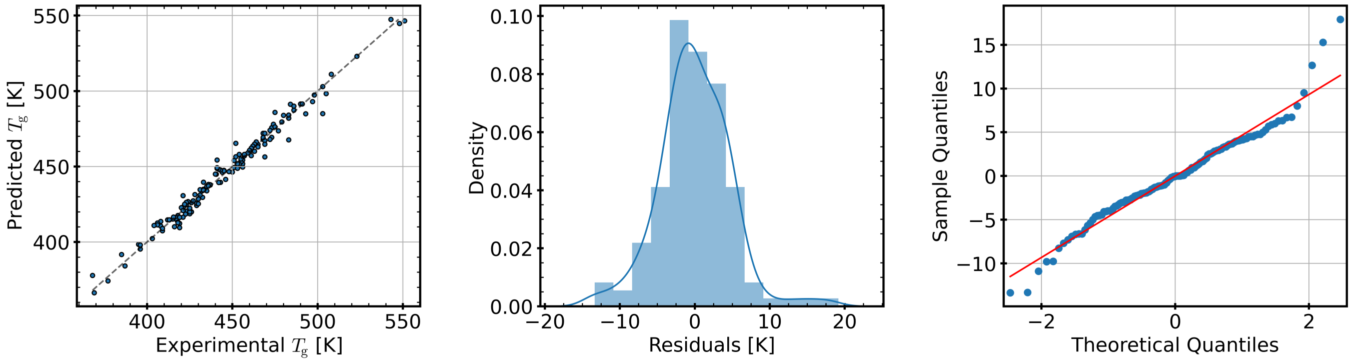

(b) **GAP model.**

Figure S16: **Diagnostic plots for assumption of normality.** Each row of figures shows the fitted against measured T_g, the distribution of residuals, and Q-Q (quantile-quantile) plots of the residuals (from left to right) for: a) QSPR-GAP GA2 model with `Mor05m` and `Mor26m`; the estimated standard deviation of the residuals is 8.5 K. b) GAP model; the estimated standard deviation of residuals is 5.2 K.

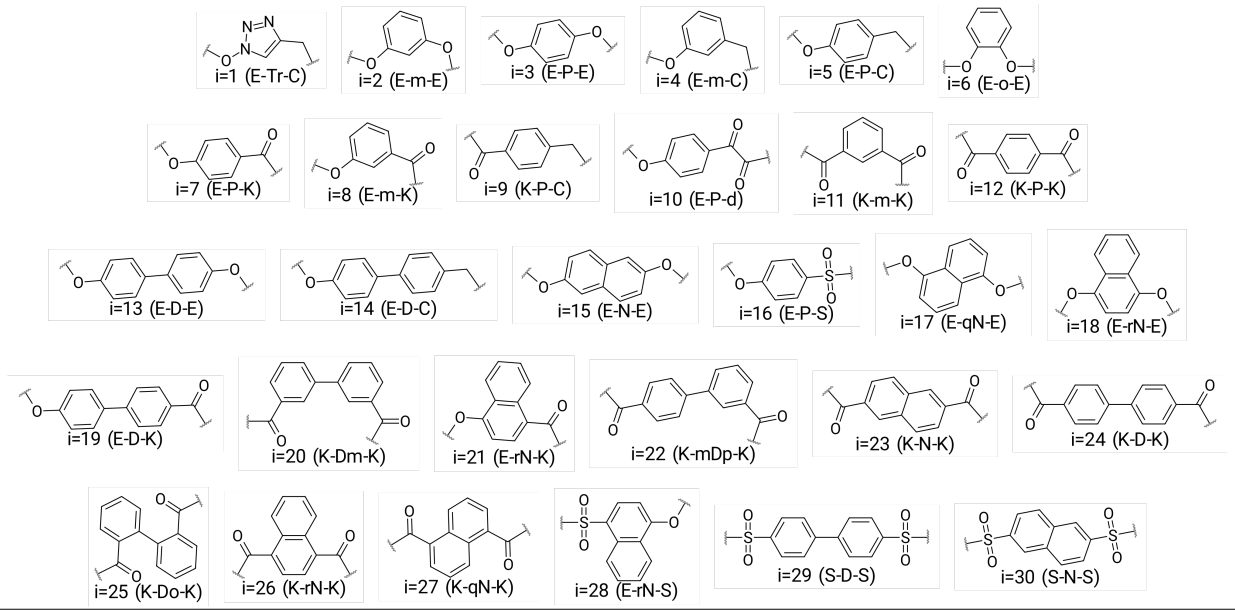

**Fragment ID**		**PCR**	**Ridge**	**Lasso**	**PLS**	**GA2 Mor05m and Mor26m**				**GAP**		**Molar mass**
Abbrv.	i	$\hat{\beta}_i$ [K]	$\hat{\beta}_i$ [K]	$\hat{\beta}_i$ [K]	$\hat{\beta}_i$ [K]	$\hat{\beta}_i$ [K]	95% CI	Mor05m	Mor26m	$\hat{\beta}_i$ [K]	95% CI	M_i [g/mol]
E-Tr-C	1	319	326	312	321	311	304/317	-0.511	0.0866	340	332/347	82
E-m-E	2	389	379	363	374	385	382/388	-1.334	-0.0459	340	311/368	92
E-P-E	3	378	385	380	377	387	384/390	-1.374	-0.0448	378	372/384	92
E-m-C	4	390	374	352	369	391	388/394	-1.392	-0.0589	331	314/348	91
E-P-C	5	371	376	375	367	396	394/399	-1.506	-0.0524	419	393/445	91
E-o-E	6	392	390	377	385	403	400/406	-1.493	-0.0899	386	357/414	92
E-P-K	7	420	420	424	422	417	415/419	-1.687	-0.1030	425	422/429	98
E-m-K	8	415	409	399	408	420	418/422	-1.702	-0.1149	393	372/414	98
K-P-C	9	411	412	426	414	425	424/427	-1.762	-0.1226	402	393/411	97
E-P-d	10	437	436	436	438	433	429/438	-2.077	-0.0708	436	428/443	112
K-m-K	11	436	434	420	427	445	443/447	-1.899	-0.1825	400	387/413	104
K-P-K	12	458	452	457	461	456	454/459	-2.007	-0.2071	466	455/477	104
E-D-E	13	478	474	471	477	469	465/472	-2.374	-0.1615	468	461/475	168
E-D-C	14	457	471	483	471	474	469/478	-2.482	-0.1551	493	479/508	167
E-N-E	15	448	464	453	450	480	478/482	-2.292	-0.2438	413	385/440	142
E-P-S	16	496	494	494	498	486	484/488	-2.381	-0.2462	497	490/504	116
E-qN-E	17	491	496	505	483	500	498/503	-2.488	-0.2887	512	493/532	142
E-rN-E	18	495	497	500	485	501	498/504	-2.472	-0.2965	508	476/541	142
E-D-K	19	502	499	500	502	505	500/510	-2.808	-0.2188	492	482/501	174
K-Dm-K	20	517	531	508	516	531	524/537	-3.084	-0.2647	520	486/553	180
E-rN-K	21	533	533	533	532	531	527/535	-2.852	-0.3359	536	530/542	148
K-mDp-K	22	509	525	510	514	533	527/539	-3.096	-0.2724	532	499/566	180
K-N-K	23	545	544	537	550	540	531/549	-2.543	-0.4726	536	524/548	154
K-D-K	24	513	515	514	509	540	534/546	-3.166	-0.2891	519	508/530	180
K-Do-K	25	509	518	507	489	546	541/551	-3.118	-0.3326	500	478/522	180
K-rN-K	26	579	574	580	590	562	557/568	-3.150	-0.4058	561	539/583	154
K-qN-K	27	560	567	566	574	564	559/570	-3.242	-0.3900	567	542/591	154
E-rN-S	28	608	592	598	593	591	584/597	-3.441	-0.4637	597	568/626	166
S-D-S	29	630	640	638	623	640	628/652	-4.398	-0.4306	646	614/679	216
S-N-S	30	654	657	652	651	690	675/705	-3.925	-0.8238	635	607/663	190

Table S5: **Table of $\hat{\beta}_i$ for all fragments and models.** The first column provides the fragment identity as shown in the figure above. Here, we tabulate the resulting $\hat{\beta}_i$ values from the following QSPR-GAP methods: Principal component regression (PCR), Ridge regression, Lasso regression, Partial Least Squares (PLS) regression, Ordinary Least Squares regression with a 2 feature subset selection via the genetic algorithm (GA2) for the two 3D-MoRSE descriptors noted. A GAP model of the form *L-Ar-L* is also presented, using OLS. Upper (UCI) and lower (LCI) 95% confidence intervals for the unbiased estimators (GA2 and GAP) are also presented. Note that the molar mass M_i of an *L-Ar-L* fragment is the molar mass of half of each L group and the full Ar group: $M_i = M_{L_{i1}}/2 + M_{Ar_i} + M_{L_{i2}}/2$.

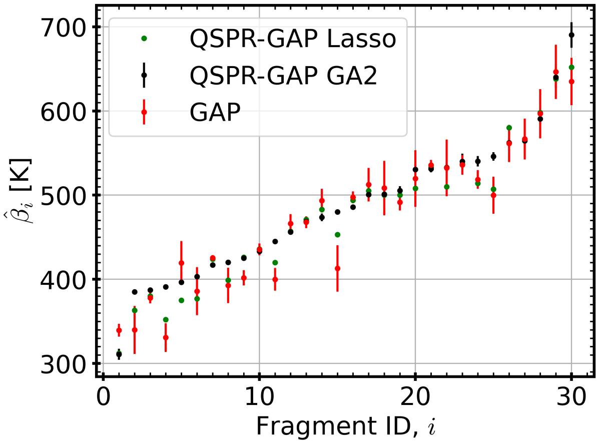

Figure S17: **Fragment T_g contributions $\hat{\beta}_i$ for Lasso, GA2 ad GAP models.** $\hat{\beta}_i$ and corresponding confidence intervals are presented as indicated in Table S5. Note here that the GAP model's confidence intervals (shown as vertical bars) are generally larger for the fragment identities that are poorly represented by the data in the fragment composition matrix; these are the the highly sparse columns in Fig. S4, such as $i = 15, i = 20$, and $i = 28$.

S-V. ATOM PAIR CONTRIBUTIONS $\hat{\pi}_{ij}$

The following results in Fig. S18 and Fig. S19 are the estimated atomic pair T_g contributions, denoted $\hat{\pi}_{kl}$ for each kth and lth atom pair. The fragments shown are a selected few from the 30 unique fragments in the dataset. The contributions were calculated from the two descriptors `Mor05m` and `Mor26m` selected by the GA (Eq. 7 in the main text). The overall fragment contribution, denoted $\hat{\beta}_i$ is determined by summing over all the $\hat{\pi}_{kl}$ values that exist in fragment i plus the constant $\hat{\gamma}_0$ (Eq. 6 in the main text). In these plots, $\hat{\pi}_{kl}$ contributions from atom pairs containing a hydrogen are ignored since these are very weak contributions.

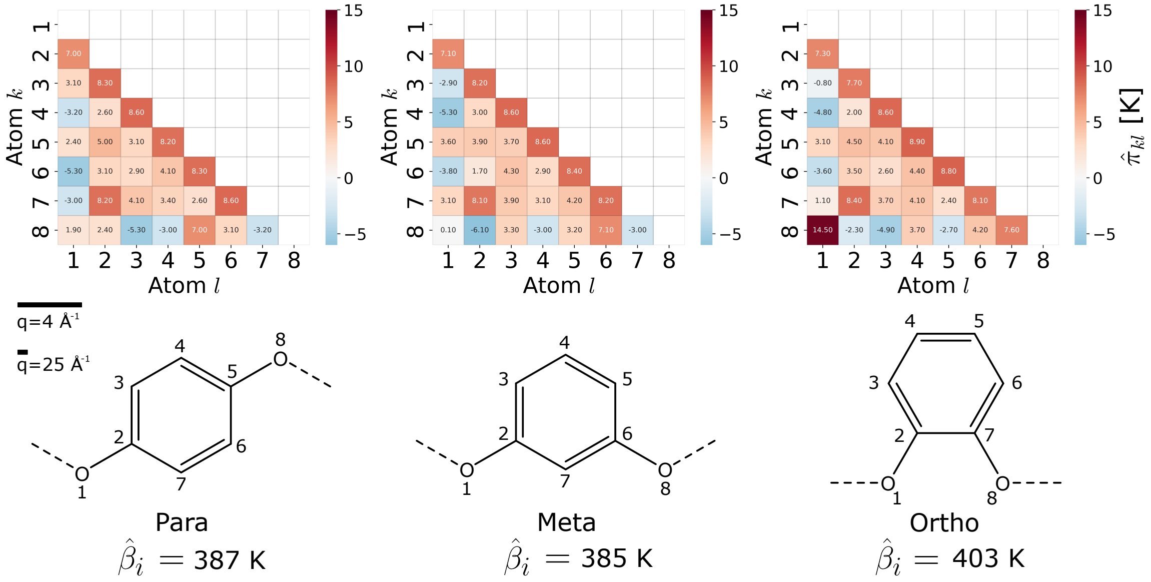

Figure S18: **Atomic pair contributions for phenylene variants.**

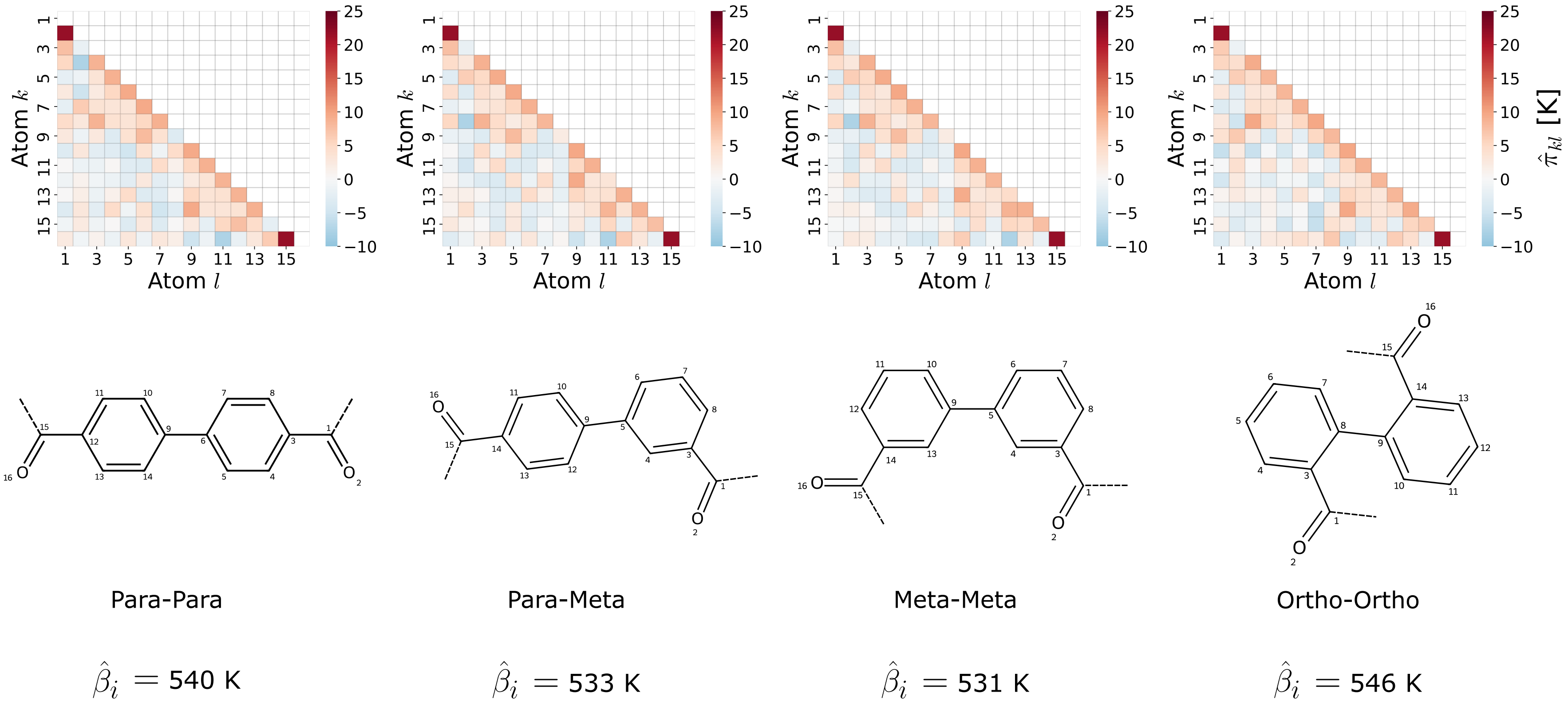

Figure S19: **Atomic pair contributions for biphenylene variants.**

S-VI. FRAGMENT DEFINITIONS

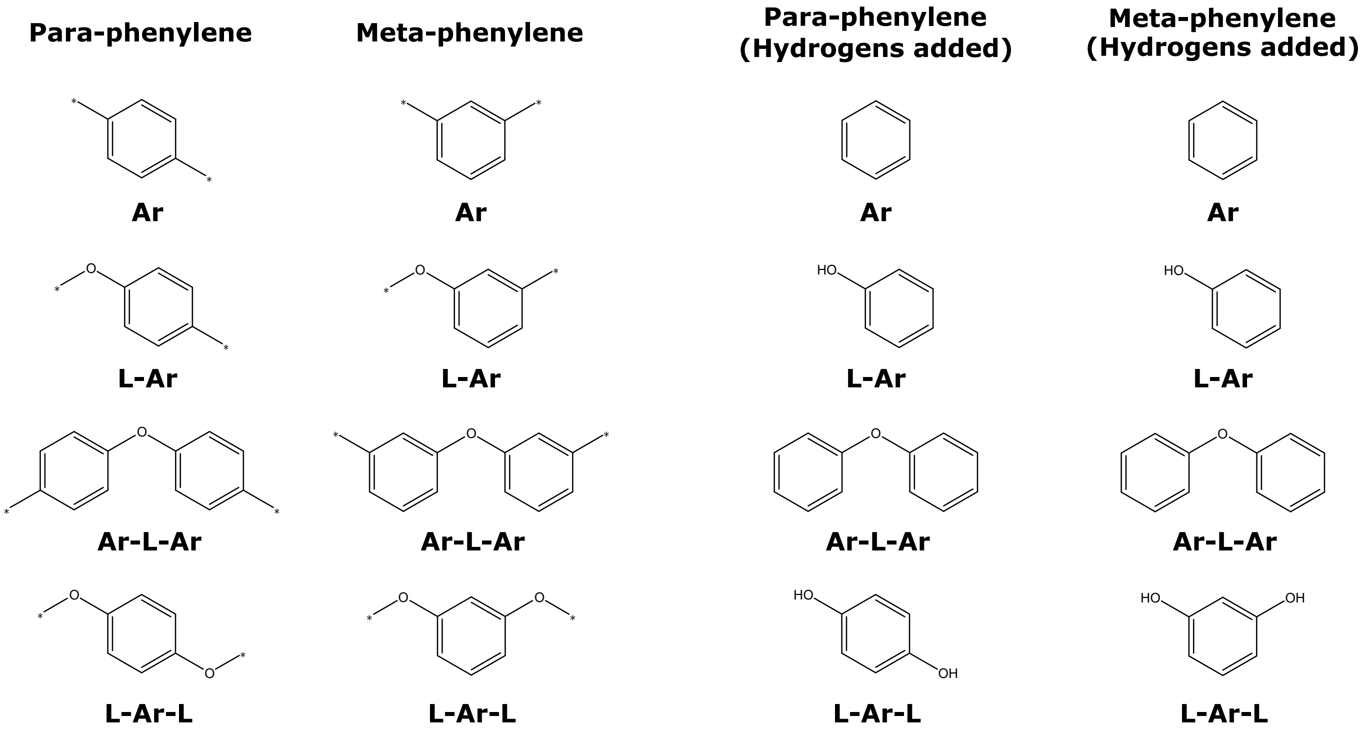

Figure S20: **Motivating the choice of *L-Ar-L*.** The figure shows two examples of phenylene variants (meta and para linked) that appear in different fragments. The connection of each fragment to its neighbours (in the backbone) is through the symbol $-*$. We must replace $-*$ in each fragment by hydrogens in order to generate their 3D descriptors. When performing this replacement for para and meta phenylenes, the only definition that uniquely distinguishes these two is the *L-Ar-L* definition. Hence, we use the *L-Ar-L* definition of a fragment.

S-VII. REFERENCES

[1] Weininger, D. SMILES, a chemical language and information system. 1. Introduction to methodology and encoding rules. *Journal of Chemical Information and Computer Sciences* **1988**, *28*, 31–36.

[2] Weininger, D.; Weininger, A.; Weininger, J. L. SMILES. 2. Algorithm for generation of unique SMILES notation. *Journal of Chemical Information and Computer Sciences* **1989**, *29*, 97–101.

[3] RDKit: Open-source cheminformatics. 2023; `https://www.rdkit.org`, doi:10.5281/zenodo.10893044.

[4] Moriwaki, H.; Tian, Y.-S.; Kawashita, N.; Takagi, T. Mordred: a molecular descriptor calculator. *Journal of Cheminformatics* **2018**, *10*, 4.

[5] Hastie, T.; Friedman, J.; Tibshirani, R. *The Elements of Statistical Learning*; Springer New York: New York, NY, 2001.

[6] Murphy, K. P. *Probabilistic Machine Learning: An Introduction*; MIT Press: Cambridge, MA, 2022.

[7] Jolliffe, I. *Principal Component Analysis*; Springer-Verlag: New York, 2002.

[8] Pedregosa, F. et al. Scikit-learn: Machine Learning in Python. *Journal of Machine Learning Research* **2011**, *12*, 2825–2830.

[9] Wegelin, J. A. A Survey of Partial Least Squares (PLS) Methods, with Emphasis on the Two-Block Case; 2000; `https://stat.uw.edu/ research/tech-reports/survey-partial-least-squares-pls-methods-emphasis-two-block-case`.

[10] Sukumar, N.; Prabhu, G.; Saha, P. In *Applications of Metaheuristics in Process Engineering*; Valadi, J., Siarry, P., Eds.; Springer International Publishing: Cham, 2014; pp 315–324.

[11] Gasteiger, J. In *Handbook of Chemoinformatics*; Gasteiger, J., Ed.; Wiley, 2003.

[12] Colquhoun, H. M.; Aldred, P. L.; Zhu, Z.; Williams, D. J. First Structural Analysis of a Naphthalene-Based Poly(ether ketone): Crystal and Molecular Simulation from X-ray Powder Data and Diffraction Modeling. *Macromolecules* **2003**, *36*, 6416–6421.
